# Supplementary figures and images for: Magnesium phosphate functionalized graphene oxide and PLGA composite matrices with enhanced mechanical and osteogenic properties for bone regeneration
Source: Regen Biomater. 2025 Jul 26;12:rbaf074. doi: 10.1093/rb/rbaf074 (PMC12364435; doi:10.1093/rb/rbaf074)

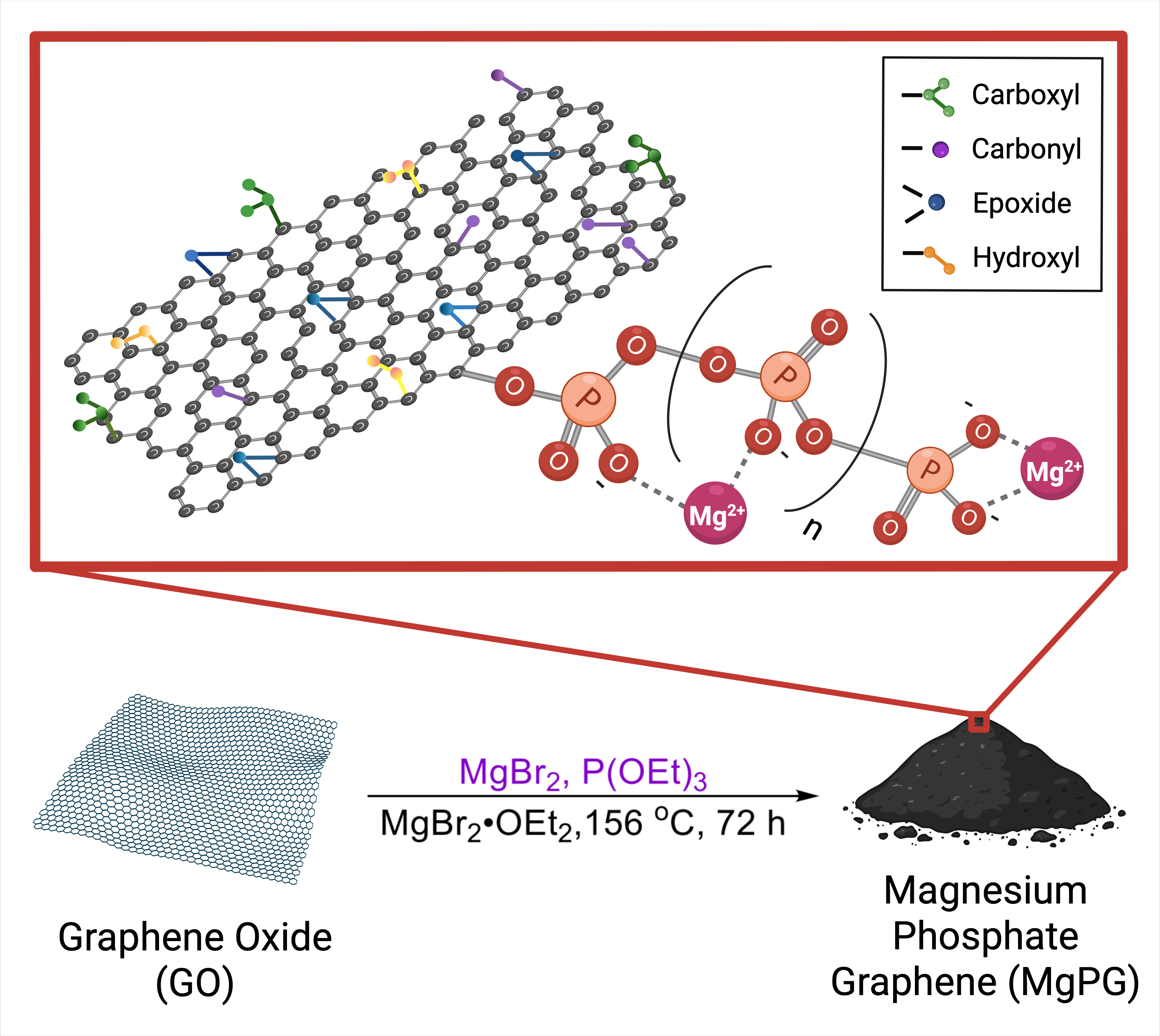

Supplement: rbaf074_Supplementary_Data [file rbaf074_supplementary_data.zip › Final_Supp_1.jpeg]
