# Supplementary material for: Magnesium phosphate functionalized graphene oxide and PLGA composite matrices with enhanced mechanical and osteogenic properties for bone regeneration
Source: Regen Biomater. 2025 Jul 26;12:rbaf074. doi: 10.1093/rb/rbaf074 (PMC12364435; doi:10.1093/rb/rbaf074)
Supplement: rbaf074_Supplementary_Data [file rbaf074_supplementary_data.zip › rbaf074_Supplement_Data.zip!/TarajeW_Regen Biomat Paper_Revised_TW(06202025) w highlights.docx]

**Magnesium Phosphate Functionalized Graphene Oxide and PLGA Composite Matrices with Enhanced Mechanical and Osteogenic Properties for Bone Regeneration**

Taraje Whitfield^1,3,6^, Fatemeh S. Hosseini^1,3,6^, Jason D. Orlando^7^, Chenyun Deng^7,8^, Kevin W.-H. Lo^1,2,4,10^, Ho-Man Kan^1^, Debolina Ghosh^1^, Stefanie A. Sydlik^7-9*^, Cato T. Laurencin^1-5*^

*^1^The Cato T. Laurencin Institute for Regenerative Engineering, University of Connecticut, Farmington, CT, 06030, USA.*

*^2^Department of Biomedical Engineering, University of Connecticut, Storrs, CT, 06269, USA.*

***^3^****Department of Orthopaedic Surgery, University of Connecticut Health, Farmington, CT, 06030, USA.*

***^4^****Department of Materials Science & Engineering, University of Connecticut, Storrs, CT, 06269, USA. USA.*

***^5^****Department of Chemical & Bimolecular Engineering, University of Connecticut, Storrs, CT, 06269, USA.*

*^6^Department of Skeletal Biology and Regeneration, UConn Health, Farmington, CT, 06030 USA*

*^7^Department of Chemistry, Carnegie Mellon University, Pittsburgh, PA, 15213, USA*

*^8^Department of Biomedical Engineering, Carnegie Mellon University, Pittsburgh, PA, 15213, USA*

*^9^Department of Materials Science and Engineering, Carnegie Mellon University, Pittsburgh, PA, 15213, USA*

*^10^Department of Medicine, Division of Endocrinology, University of Connecticut Health Center, School of Medicine, Farmington, CT, USA*

****Corresponding Authors:***

*Cato T. Laurencin, M.D., Ph.D.*

*E‐mail:* [*laurencin@uchc.edu*](mailto:laurencin@uchc.edu)

*Stefanie A. Sydlik, Ph.D.*

*Email:* [*ssydlik@andrew.cmu.edu*](mailto:ssydlik@andrew.cmu.edu)

**Competing Interest Statement:**

The authors have no relevant financial or non-financial interests to disclose.

**Keywords:**

Magnesium Phosphate, Graphene Oxide, Bone Regeneration, Wnt/β-Catenin, Induceron

**This File Includes:**

Graphical Abstract

Figures 1-6

Table 1

**Abstract**

Bone defects affect millions of people annually, making bone tissue of particular interest for developing treatments. Current strategies for healing suffer drawbacks. Regenerative engineering seeks to achieve efficient bone regeneration by utilizing synthetic bone grafts to evade these drawbacks. One material that offers such benefits is a class of functional graphenic material, known as Phosphate Graphenes. While many of our studies have focused on Calcium Phosphate Graphene, magnesium is also osteogenic. Therefore, in this study, we utilized regenerative engineering techniques to incorporate Magnesium Phosphate Graphene (MgPG) into poly(lactic-co-glycolic acid) (PLGA) to fabricate composite microsphere-based matrices as a potential synthetic bone graft. Employing different amounts of MgPG within PLGA matrices, we studied the effect of MgPG on the morphological, structural, physical, and biological characteristics. MgPG-containing matrices demonstrated great mechanical strength, hydrophilicity, and degradability without compromising matrix morphology. Because MgPG is a graphene oxide derivative with magnesium and phosphate ions capable of supporting bone healing as inducerons, we next evaluated the cytocompatibility and osteogenic potential of these PLGA/MgPG composite matrices. MgPG matrices demonstrated high cell viability and proliferation of MC3T3-E1 cells as well as increased osteogenic activity reported by alkaline phosphatase activity, calcium deposition, and gene expression of Col1a1, osteocalcin, bone sialoprotein, and Sp7. Lastly, we investigated the gene expression profile of markers/targets of the canonical β-catenin dependent Wnt signaling pathway with and without inhibitor DKK1 to understand the potential underlying mechanism behind the enhanced osteogenic potential of MgPG. In response to MgPG, gene expression of β-catenin increased, while protein expression of BMP-2 and WISP-1 also increased. These results suggest the influence of MgPG on the Wnt pathway in relation to osteogenic differentiation. With further study, MgPG matrices may provide practical solutions to the problem of effectively regenerating critical-sized bone defects, which remains a challenge in orthopaedics.

**Graphical Abstract**

**Main Text**

**Introduction**

Millions of patients experience bone injuries each year [1]. Many of these injuries are classified as critical-sized bone defects, meaning they will not heal independently and require external intervention, which is a major problem faced by orthopaedic surgeons in particular [2]. Specifically, these defects are defined as being at least twice the length of the diaphysis diameter for long bones, but the “critical” nature of the defect is also dependent upon factors such as anatomic location of the defect, the damage to the soft tissues surrounding it, age of the patient, and the presence of chronic disease and other comorbidities [3, 4].

The current gold-standard treatment for such defects is an autologous bone transplantation, coming predominantly from the host’s iliac crest. Unfortunately, such a treatment has the drawbacks of limited tissue amount, risk of co-morbidity at the donor site, and risk of postoperative infection [5]. Other treatment options include other autografts, allograft, xenograft, or metallic implants, each of which has many drawbacks [6]. Autografts require a secondary surgery to extract a limited amount of donor tissue; allografts/xenografts may lead to transplant rejection; and metallic implants lack osteointegration, keeping them permanently in the body with toxic corrosion overtime [6].

Regenerative engineering aims to provide a practical answer to these problems for all tissues via bioengineered synthetic grafts [7-31]. To successfully regenerate bone tissue, various engineering approaches are being developed in the regenerative engineering, including 3D-printing, microsphere use, fiber braiding, electrospinning, electrospraying, casting, and molding [32-39]. With these techniques, an ideal matrix for bone regeneration acquires several key properties to effectively support tissue repair and regeneration. The synthetic bone graft must be mechanically competent, biocompatible, biodegradable, osteoconductive, and osteoinductive [40]. Despite decades of research attempting to encompass all these characteristics through these various techniques, a synthetic bone graft that can replace current therapies has yet to be achieved [41, 42].

Graphene-based materials have shown promise as synthetic bone grafts. Graphenic materials are promising candidates due to their exceptionally high mechanical strength, bioresporbabaility, chemical tailorability, and high surface area [43-48]. The most common type of graphene material used in biomedical applications is graphene oxide (GO), a derivative of graphene. As the name suggests, GO is a highly oxidized form of graphene that contains oxygen functional groups (such as hydroxyl, carboxyl, and epoxy groups) that enable further functionalization and attachment of useful biomolecules [49-51]. Additionally, these oxygen-rich functional groups exhibit protein adsorption [52], cell attachment and proliferation platforms [53], Ca^2+^ ion adsorption, and enhanced interaction with the matrix for increased load transfer efficiency [54]. GO has also been shown to promote osteogenic stem cell differentiation [52, 55] and enhance the expression of early and late osteogenic markers such as RUNX-2, BMP-2, osteocalcin (OCN), osteopontin (OPN), and Col1a1, respectively [56]. Therefore, GO sheets can be used as a bioactive platform to enhance and accelerate cell adhesion, proliferation, and differentiation through protein adsorption and provide a Ca^2+^ nucleation site that enhances hydroxyapatite formation [57]. By combining its excellent mechanical properties (high strength, flexibility, stiffness, etc.) with its promising biochemical properties, GO and its derivatives are promising materials to be used with biomaterials for the fabrication of a synthetic bone graft.

Another difficulty in the development of an ideal bone graft is that while most are osteoconductive, there are no current Food and Drug Association (FDA) approved synthetic bone grafts on the market that are osteoinductive, highlighting the need to fabricate a graft with the capabilities of directing stem cell differentiation toward osteoblasts [58, 59]. Due to this lack of osteoinduction, often times, currently available synthetic bone grafts are used in conjunction with various growth factors to achieve the desired osteoblastic stem cell differentiation. While growth factors play a critical role in the regulation of various cellular processes involved in bone formation, their clinical application has been constrained by several limitations such as cancer development and ectopic tissue formation, limited by issues related to stability, cost, and potential side effects [60]. It is also challenging to deliver growth factors locally and sustainably at the target site, which often requires high doses and frequent administrations to achieve therapeutic success [60]. These drawbacks have prompted researchers to investigate alternative strategies, such as the utilization of "inducerons", or ions that are known to direct osteogenic differentiation of stem cells and causing them to synthesize their own growth factors, eliminating the use of exogenous growth factors [60]. Inducerons such as calcium and phosphate have been shown to activate signaling pathways involved in bone regeneration and simulate bone tissue mineral composition, thereby inducing osteogenic differentiation of stem cells without exogenous growth factors [61, 62]. Other osteogenic inducerons include boron, cobalt, copper (II), fluoride, lithium, niobium, silicate, silver, strontium, vanadium, zinc, and magnesium [63].

The replacement of growth factors with materials that combine inducerons is evident by the field’s growing use of magnesium phosphates—mineral compounds. Magnesium containing scaffolds have recently been reported for use in bone grafts [64] and show great potential for therapeutic applications in bone regeneration [65-67]. As magnesium phosphate-based matrices demonstrate high initial strength, hydrolytic degradation potential, and enhanced regenerative properties, their use as bone grafts has increased [64, 68, 69]. Recent research has shown that magnesium phosphate can promote bone regeneration by stimulating the activity of osteoblasts, the cells responsible for forming new bone tissue, both *in vitro* and *in vivo* [70-72]. Furthermore, at the molecular level, the Wnt pathway is a key regulator of bone healing and the regeneration processes, as it regulates various aspects of skeletal development, including bone formation, remodeling, and repair. The Wnt pathway is critical for regulating proliferation, differentiation, growth, survival, development, regeneration, self-renewal, and osteoblastogenesis to promote bone formation/healing, which all act in opposition to osteoclasts which resorb bone tissue [73]. In concurrence with the induceron approach, materials that release Mg have been shown to activate the canonical Wnt signaling pathway [74-80], as well as those that contain phosphates [81-83]. Similar to the inducerons, GO has also been highly cited as an inducer of the Wnt pathway for bone regeneration [84-87].

The remarkable osteogenic properties of these three materials (graphene oxide, magnesium, and phosphate) led to the combination of them all into a single material for use as a bone graft. Our groups previously reported the novel synthesis method of phosphate graphene (PG) utilizing the Arbuzov reaction via a Lewis acid catalyst [61]. The reaction conditions allow for remarkable customization of PG composition, resulting in the synthesis of a hydroxyapatite-like surface by incorporating polyphosphate groups on GO with innate osteoinductivity, robust mechanical strength, an aqueous degradation pathway, bioactive cell attachment interfaces, mineralization platforms due to calcium adsorption, and a tunable surface chemistry [88, 89]. Furthermore, this chemistry allows for the inclusion of different counterions (calcium, potassium, lithium, magnesium, or sodium) to be added to the phosphate functioned GO, leading to their eventual controlled release [61]. The selection of magnesium as a counterion for PG constituted the generation of Magnesium Phosphate Graphene (MgPG) to be used as a bone graft material, which may evade all the drawbacks associated with the current state of bone regeneration. MgPG is a powder, however, so it cannot be delivered into patients as a self-stabilizing osteogenic scaffold currently (Supplementary Figure 1).

To address the deliverability issue of these materials, our groups demonstrated that the FDA-cleared polylactic-co-glycolic acid can be a promising carrier for MgPG [90]. In our previous work, GO has been studied in PLGA microsphere-based matrices at concentrations of 0 – 10 weight percentage (wt%) with 5wt% demonstrating the strongest osteogenic responses [90]. These matrices were not only able to package GO into a ready-to-use scaffold, but they also demonstrated great bone matrix characteristics [90]. Therefore, we hypothesized that similarly incorporating Magnesium Phosphate Graphene (MgPG) into PLGA-based synthetic bone matrices will enhance the osteogenic properties of the matrix *in vitro* by releasing inducerons that stimulate the Wnt pathway in MC3T3 cells. In this study, we successfully fabricated microsphere-based PLGA/MgPG composite matrices for bone regeneration. Furthermore, we assessed the cytocompatibility (via live/dead staining and MTS assay); and the osteogenic potential (via alkaline phosphatase activity and alizarin red staining) of the PLGA/MgPG composite matrices *in vitro.* Lastly, we investigated the interaction of MgPG with MC3T3 cells to elucidate the underlying gene/protein-related mechanisms of enhanced osteogenic activity via the Wnt pathway.

**Results**

**Classifications**

To fabricate a bioactive matrix for bone regenerative engineering, microspheres were synthesized according to the following five study groups: PLGA (100% PLGA), PLGA/GO1 (99% PLGA + 1% by weight GO), PLGA/GO5 (95% PLGA + 5% by weight GO), PLGA/MgPG1 (99% PLGA + 1% by weight MgPG), and PLGA/MgPG5 (95% PLGA + 5% MgPG) (Figure 1A). Upon synthesis, the microspheres were heat sintered into matrices, successfully completing the bioengineering fabrication process.

**Magnesium Phosphate Graphene Matrix Properties**

To understand the properties of our bioengineered matrices, several characterization tests were run. MicroCT analysis on the full matrix allowed us to visualize all aspects of the matrix 3-dimensionally, including the size of the microspheres which fell within their expected range of 300 – 600 μm in diameter for all groups with no significant differences between them (Figure 1B-C). Previous studies from our group have shown that this microsphere size range created the desired pore sizes and pore volumes within the matrices for cellular interactions [91]. Furthermore, the use of 300 – 600 μm microspheres allowed for a consistent pore size of ~290 μm between groups, as expected, showing no significant differences among them (Figure 1D). This pore size, on the scale of 10 times larger than mesenchymal stem cells (15 – 30 µm) is critical for cell infiltration [92].

**Figure 1: Magnesium Phosphate Graphene Matrix Properties**
(A) Schematic of a PLGA/MgPG composite microsphere including its chemically added ions for release in aqueous environments. (B) The 3-Dimensional µCT image of a PLGA/MgPG composite matrix (C) Microsphere diameter measurements as determined by µCT analysis showing no difference in microsphere size as a result of sieving for microspheres within the range of 300 - 600 μm (D) Measurements of matrix pore size as determined by µCT analysis revealing that a consistent pore size was maintained among groups as the microsphere size was also held constant. (E) Average weights of 7x1 mm matrices.

**Morphological Characterization of Composite Matrices**

After obtaining matrices with the proper microsphere and pore sizes, we next analyzed the microstructure and morphology of the microspheres and the microsphere-based matrices. To do so, we used SEM, which showed that the gross appearance and surface morphology of the PLGA and MgPG groups demonstrated a round and smooth exterior surface similar to PLGA as the control group (Figure 2A). SEM further showed that as the amount of MgPG increased within the microspheres, there was no difference among the study groups of their exterior roughness and abundance of pores.

Switching from external examination to internal, we then analyzed the MgPG powder itself as well as the interior of the microspheres by cutting them in half before imaging (Figure 2B). Pure MgPG powder demonstrated a layered appearance from the stacked GO sheets via SEM, as similar amounts of magnesium (blue) and phosphorus (magenta) (6 and 7 atomic percentage of MgPG, respectively) from the chemical addition of magnesium and phosphate via EDS mapping (Figure 2B, Supplementary Figure 2, Supplementary Table 1). Within PLGA/MgPG1 and PLGA/MgPG5 microspheres, SEM/EDS mapping also labelled magnesium and phosphorus embedded within the PLGA (Figure 2B). Due to the initial phosphate graphene studies done by Sydlik et al. (namely FTIR, XPS, and phosphate quantification and elution assays), we can confidently conclude that phosphorus within our materials is in the phosphate state [61]. Therefore, these images visually revealed the compositions of the microspheres/scaffolds while also showing a uniform dispersion of MgPG powder and its expected elements through the PLGA microspheres.

**Figure 2: Morphological Characterization of Composite Matrices.**
(A) Scanning Electron Microscopy (SEM) confirmed a smooth surface of the microspheres and matrices. Top row shows one singular microsphere from each group. Bottom row shows a zoomed in area of the heat sintered microsphere. The flat parts of the microspheres are where the spheres were in contact with the top of the metallic sintering mold. (B) Energy Dispersive X-Ray Spectroscopy (EDS) displays the distribution of Mg ions within the MgPG powder and the cut-in-half PLGA/MgPG microsphere hemispheres.

**Material Characterization of the Composite Matrices**

Next, we verified that the matrices were chemically consistent with our observations of successful incorporation of MgPG at 1 and 5 wt% into PLGA as well. To study the encapsulation of MgPG sheets within the composite spheres, thermogravimetric analysis (TGA) was used to assess the presence and quantify the amount of MgPG encapsulated within the polymeric spheres (Figure 3A-B). This was possible because of the differing decomposition temperatures of PLGA and MgPG. The structural decomposition of PLGA initiated at ~230℃, demonstrating a sharp drop in mass corresponding to burning of the organic component and completed at ~400℃. All composite samples demonstrated this pronounced mass loss, which is consistent with PLGA being the major constituent of the composite spheres. As the temperature increased beyond 400℃, the remnant mass stabilized, with only trace amounts of final residue remaining at ~590℃. The pure powder of MgPG demonstrated higher thermal stability, revealing 19.2% weight loss at 590℃ before stabilizing. Utilizing the differences in thermodynamic stabilities of the powder vs polymer, the residual mass of the composite microspheres stabilized around 5% and 1%, indicating the presence of the expected amounts of MgPG present within our microsphere-based matrices. Upon complete burning of the samples up to 800℃, the remaining residual mass percentages were used to calculate the efficiency of the oil-in-water emulsion solvent technique at encapsulating the MgPG powder within PLGA microspheres. The encapsulation efficacies of PLGA/MgPG1 and PLGA/MgPG5 were calculated as 85.09% and 91.96%, respectively (see Supplementary Data for calculations).

After demonstrating the successful incorporation of the MgPG powder into PLGA, our next goal was to understand the dispersion state of the incorporated powder. To determine the chemical make-up and structure of the matrices, X-Ray powder Diffraction (XRD) analysis was performed (Figure 3C). The XRD spectra for all groups demonstrated amorphous behavior (by curved and undefined peaks) rather than crystalline, which would have been shown by clear, distinct, sharp peaks. Although GO is crystalline, it made up only a small percentage of the composite microspheres, which were predominantly PLGA—an amorphous polymer with a diffraction peak of 20° [93]. The incorporation of graphenic powders did not change the x-ray diffraction behavior of PLGA.

The chemical composition of the PLGA/MgPG5 composite microspheres was characterized using Fourier Transform Infrared Spectroscopy (FTIR) to identify characteristic vibrational modes associated with both PLGA and MgPG components (Figure 3D). In the 2800–3100 cm⁻¹ region, peaks consistent with sp³-hybridized C–H stretching vibrations from methylene (CH₂) groups are observed, supporting the presence of alkyl chains within the MgPG filler. In some samples, these peaks are obscured by a broad hydrogen bonding signal, or lower sample signal. In the carbonyl region (1650–1800 cm⁻¹), both PLGA and MgPG exhibit strong C=O stretching peaks, confirming the presence of ester or carboxylic functionalities in the composite. The fingerprint region reveals subtle but distinct differences between the spectra of GO (purple) and MgPG (green), with MgPG showing additional phosphate-related features. Notably, the FTIR spectra of PLGA/MgPG5 closely resemble that of pure PLGA, though small shifts and intensity changes in these key regions indicate successful incorporation of MgPG. Upon thermal removal of the PLGA component (gold trace – PLGA/MgPG5 w/o PLGA), the remaining spectrum matches that of the pure MgPG powder (green), further verifying the presence and chemical identity of MgPG in the composite microspheres.

**Figure 3: Material Characterization of the Composite Matrices.**
(A) Thermogravimetric Analysis (TGA) confirming the incorporation of MgPG into the PLGA-based microspheres at their proper weight percentages of 1 and 5 wt% as PLGA burned out of the matrices, while only the incorporated amounts of MgPG powder remained. The corresponding encapsulation efficacies of MgPG powder incorporation into PLGA microspheres were also calculated and can be found in the supplementary data. (B) TGA derivative weights confirming the TGA data. (C) X-Ray Diffraction analysis (XRD) revealed the semi-crystalline/semi-amorphous nature of the PLGA/MgPG composite microspheres. Pure PLGA had a broad X-Ray diffraction peak around 20°, which remained present with the incorporation of graphene oxide and magnesium phosphate graphene at 1 and 5 wt%. (D) Fourier Transform Infrared (FTIR) spectra of all materials, particularly PLGA/MgPG5 microspheres after the thermal removal of PLGA (PLGA/MgPG5 w/o PLGA), confirmed that MgPG was, indeed, incorporated into PLGA microspheres without significantly altering its chemical nature. Differences in MgPG from GO due to magnesium phosphate functionalization were also revealed.

**Physical Properties and Characteristics of the Composite Matrices**

After confirming the morphological and chemical fabrication of matrices, we lastly sought to study the physical properties and characteristics of our bioengineered matrices.

Uniaxial compressive tests were performed on matrices of 5 mm thickness and 5 mm diameter to evaluate the mechanical properties of matrices and the effects of the incorporation of MgPG and GO (Figure 4A-C). Figure 4A shows the stress-strain curves of all groups. From six samples from each group, the most representative sample of the group was graphed, showing that the addition of graphenic materials to PLGA allowed the matrix to stay intact under high load (up to 50% strain), whereas PLGA on its own failed at 9.40 ± 1.74% strain. Quantification of the stress-strain curve information showed that the addition of graphenic material to PLGA also caused a significant increase in strength, whether it was GO or MgPG at either 1 or 5 wt%. Most intriguing, PLGA/MgPG5 was four times stronger than PLGA alone. Additionally, PLGA/MgPG at 1 and 5 wt% (36.44 ± 1.41 MPa and 38.81 ± 0.99 MPa, respectively) were significantly stronger than PLGA/GO1 (28.79 ± 3.41 MPa), and PLGA/MgPG5 (38.81 ± 0.99 MPa) was significantly stronger than PLGA/GO5 (33.64 ± 4.95 MPa), implying that the addition of the magnesium and phosphate ions onto the backbone of GO provided additional strength to PLGA than GO did on its own (Figure 4B). Finally, although the matrices displayed different ultimate strengths, all compressive moduli values for all groups were not significantly different from each other, showing that the matrices are very similar in terms of hardness (Figure 4C).

Functional graphenic materials enhance substrate hydrophilicity, which influences biocompatibility and the interaction between graphene-containing matrices and the surrounding cells [94]. To measure these properties in our matrices, various static contact angles were measured to determine the wettability and hydrophilic/hydrophobic characteristics of the matrices (Figure 4D). Hydrophilic properties were demonstrated by PLGA's 82.53 ± 2.40° water contact angle, but in the presence of GO and MgPG, the water contact angle decreased significantly, displaying increased hydrophilicity. Although there were no significant differences between the graphenic material groups, all groups were significantly lower than PLGA, revealing that the integration of graphenic material corresponded with an increase in hydrophilicity of bioengineered matrices.

The biodegradation behavior of matrices was studied to assess their potential for regenerative engineering applications. To regenerate a bone effectively, the degradation of the bone matrix must match the rate of bone regeneration to enable proper healing. Over a period of 90 days, we placed our matrices in a 15 mL PBS solution for incubation to track degradation. Following the monthly incubation time points, the swelling rate percentage and weight loss percentage values were carefully determined and recorded (Figure 4F-G). In similar fashion to the results obtained from the measurements of contact angles, the addition of GO and MgPG (at 5 wt%) led to increased degradation rates relative to PLGA (except for PLGA/MgPG1) at 30 days (Figure 4F). Additionally, there was an increase in swelling rates with the addition of graphenic materials at 5 wt% compared to matrices made from pure PLGA at day 90 (Figure 4G). PLGA/GO1 and PLGA/GO5 matrices exhibited the greatest water uptake as a function of time, followed by PLGA/MgPG5. Although the incorporation of graphenic material significantly increased the swelling rates of the matrices, it is important to note that over the course of three months, the maximum average swelling did not exceed 12% for PLGA/GO matrices nor 10% for PLGA/MgPG matrices which is low.

PLGA, composed of polylactic acid (PLA) and polyglycolic acid (PGA), produces acidic byproducts upon degradation, particularly when the PLA:PGA ratio decreases, which also accelerates the degradation rate [95, 96]. Similarly, both GO and MgPG have displayed acidic byproduct release upon degradation [89]. Therefore, to ensure our bioengineered matrices would not create a harmful acidic environment within the healing bone defect, we also measured the pH of the solution our samples were degrading (Figure 4E). Relative to a control of PBS with no matrix, the pH of the environment surrounding the degrading implants did not significantly vary over 3 months.

**Figure 4: Physical Properties and Characteristics of the Composite Matrices.**
(A) Stress-Strain curve for all groups using a cylindrical 5*5 mm matrix. The most representative samples are pictured on the graph (n=6). The zoomed in window shows the initial slopes of the stress-strain curves. (B) Ultimate Compressive Strength as determined by the maximum load at failure, or the maximum load at 50% strain. (C) Compressive Modulus calculated using the 0.2% strain offset linear slope method (D) Water contact angle measurements from a 5 µL droplet on a thin film of material on a glass slide. The incorporation of MgPG into PLGA matrices led to a decrease in the water contact angle, revealing increased hydrophilicity. (E) Continual measurement of pH demonstrated that the pH was not significantly altered from a PBS control as the matrices degraded. (F) Percent weight loss measurements over the course of a 3-month period revealed a similar degradation pattern for PLGA/MgPG1 and PLGA, though MgPG at 5 wt% in PLGA increased the degradation rate without causing too much degradation. (G) Swelling rates of MgPG matrices were also significantly increased relative to PLGA at month 2 and 3 without causing excessive swelling. Quantitative experimental data were presented as mean ± standard deviation (SD). Significant levels are determined at *p < 0.05, **p < 0.01, ***p < 0.001, and ****p < 0.0001.

**Cytocompatibility and Osteogenic Potential**

After confirming the successful fabrication of matrices that can be used for bone regenerative engineering, we next sought to study their cytocompatibility and osteogenic potential. We began this process by first calculating how much magnesium, in theory, the seeded cells would encounter. The TGA showed that, indeed, 1 and 5% of the microspheres were composed of the MgPG powder with high efficacy (Figure 3A-B, Supplementary Data). Furthermore, Figure 1E shows that PLGA/MgPG1 and PLGA/MgPG5 scaffolds weighed about 48.7 and 64.9 mg, respectively. Therefore, 0.5 and 3.2 mg of the respective matrices were composed of MgPG powder. Moreover, our data showed via XPS that the MgPG powder has an elemental magnesium composition of 5%, implying that each of our matrices, in theory, released approximately 0.02 and 0.16 mg of magnesium, respectively (mg of powder * 0.05) (Supplementary Figure 2). This amount of Mg corresponds to a 1.0 and 6.7 mM concentration of magnesium ions for the subsequent *in vitro* experiments ((mg of magnesium / molar mass of magnesium in mg) / 1 mL of media). All calculations can be found in the Supplementary Data.

**Cytocompatibility**

To analyze the cytocompatibility of our matrices qualitatively, a Live/Dead assay was performed (Figure 5A). Over the course of two weeks, cells were seeded on our matrices, and from the amount of calcein (green) label, we were able to visualize the quantity of live cells within our matrices, as calcein permeates through intact cell membranes to stain live cells. On the flipside, ethidium homodimer-1 (red) label stains dead cells, as it is a protein that is highly positively charged, disabling it from entering the cell membrane while still being able to bind to DNA, targeting the DNA in dead cells that have disrupted plasma membranes. Qualitatively across all groups, live cells were readily visible using the Live/Dead assay (ThermoFisher), showing that our matrices were cytocompatible. Furthermore, with the incorporation of graphenic material, the number of cells appeared to be higher than it was for PLGA, showing increased cell viability. Furthermore, the number of live cells in the MgPG groups as opposed to GO groups also appeared to be higher, signifying that the magnesium and phosphate groups added to GO further supported cell survival and viability.

To further study cellular cytocompatibility, the quantitative MTS assay was performed in conjunction with the previously obtained Live/Dead qualitative data. The MTS results corroborated the Live/Dead data by quantitatively measuring the proliferation and metabolic activity of MC3T3 cells seeded on our matrices (Figure 5B). This assay showed that all groups containing graphenic materials were comparable to PLGA with no significant differences regarding cell growth and proliferation.

**Figure 5: Cytocompatibility and Osteogenic Potential.**
(A) Live/Dead assay showed live cells labelled with calcein (green) and dead cells with ethidium homodiomer-1 (red) revealing that MgPG scaffolds supported cell growth and survival in a manner similar to that of PLGA. Scale bar is 100 μm. (B) MTS assay measurements of the metabolic activity of MC3T3 cells showed that all matrices containing graphenic materials were comparable PLGA alone across a two-week period. (C) Alkaline Phosphate activity assay signifying bone biosynthesis across a two-week period demonstrated significantly higher amounts of ALP activity and therefore higher osteogenic potential of PLGA/MgPG5 matrices relative to PLGA, particularly at days 5, 10, and 14 in osteogenic media. (D) The Alizarin Red S assay (performed in osteogenic media) showed the quantification of calcium deposition within the bone matrices for 14, 21, and 28 days. MgPG at 1 and 5 wt% demonstrated significantly higher levels of calcium deposition at D28 than PLGA, as well as their respective GO counterparts without magnesium and phosphate. Quantitative experimental data were presented as mean ± standard deviation (SD). Significant levels are determined at *p < 0.05, **p < 0.01, ***p < 0.001, and ****p < 0.0001.

**Osteogenic Activity**

After showing the cytocompatibility of our matrices, we next sought to evaluate the osteogenic potential, since the goal of this thesis is to fabricate a matrix that supports adequate osteogenesis to be used for bone regeneration.

To understand the osteogenic potential of our matrices, we ran an alkaline phosphatase (ALP) assay which exhibited an increased amount of ALP activity (normalized to the amount of protein in the cells’ lysates) in response to MgPG-containing matrices over the course of two weeks (Figure 5C). Furthermore, as the MgPG content increased in the matrix from 1 to 5 wt%, there was a significant increase in ALP activity at D14 of growth/differentiation, demonstrating that an increase in the amount of MgPG to our bioengineered matrices led to an increase in the osteogenic potential of the matrices, surpassing that of even PLGA/GO5. More specifically, PLGA/MgPG5 appeared to be the most osteogenically favorable group in terms of secreted ALP activity, proving itself to be significantly higher than PLGA at days 10 and 14 in osteogenic media.

Next, we conducted a bone mineralization assay (ARS) to further understand the osteogenic potential of our matrices, as ARS is a more direct indicator of bone-forming potential. Mineralized matrix synthesis was assessed using the Alizarin Red S staining method for identifying calcium deposition (Figure 5D). We decided to perform this assay under normal (growth media) conditions (Supplementary Figure 3) and osteogenic media conditions (Figure 5D) to investigate MgPG’s ability to induce differentiation and calcification without osteogenic factors given that there was no significant difference in the growth/proliferation of MC3T3 cells on the MgPG-containing matrices vs those without MgPG up to 14 days. As early as day 14, however, PLGA/GO5 and PLGA/MgPG5 were able to promote an early significant increase in mineralization activity demonstrating the osteogenic effects of graphenic material inclusion. Furthermore, at Day 28, PLGA/MgPG5 demonstrated almost twice as much calcium deposition into the matrix as PLGA alone (Figure 5D). Interestingly, at Day 21 under growth media conditions, only PLGA/MgPG5 was also able to significantly increase mineralization potential when compared to PLGA matrices in the absence of external osteogenic factors present within osteogenic media (Supplementary Figure 3).

**Evaluation of the Wnt Pathway and Osteogenesis**

An important part bone regeneration is understanding the molecular mechanisms behind osteogenesis. To understand why our PLGA/MgPG materials were enabling osteogenesis, we conducted a RT-qPCR to study the Wnt pathway using β-catenin, a necessary molecule for the pathways, under normal and Wnt-specific inhibitory conditions. Additionally, we studied the gene expression of early and late osteogenic markers under the same conditions to further understand the relationship between MgPG and its effect on osteogenesis through the Wnt pathway. Studies were performed with the control, PLGA, and PLGA/MgPG5 matrices, as they demonstrated the highest extent of enhanced osteogenic activity.

At 7 days post-cell attachment, under normal basal media conditions, PLGA/MgPG5 matrices promoted a significant increase in gene expression of β-catenin, signifying an enhanced activation of the pathway (Figure 6A). Both groups displayed a decrease in their gene expression in response to DKK1, however, demonstrating the Wnt pathway specificity of DKK1. Furthermore, PLGA/MgPG5 matrix integration led to significantly higher expression profiles for SP7, bone sialoprotein (BSP), Col1a1, and OCN at day 7 (Figure 6B). With collagen 1, an important component of bone, being more than doubled in gene expression alongside various other osteogenic markers, we continued our evaluation of PLGA/MgPG5’s osteogenic abilities.

**Figure 6: Wnt and Osteogenic Expression Assays**
RT-qPCR and ELISA results after culturing MC3T3 cells on PLGA/MgPG composite matrices for 7 days in normal media and inhibitory DKK1 conditions. (A) PCR for Wnt pathway markers with and without DKK1. (B) PCR for osteogenic genes under normal media conditions (C) ELISA of Wnt-pathway target WISP-1 with and without DKK1 (D) ELISA of osteogenic markers in the presence and absence of Wnt-pathway inhibition by DKK1. Quantitative experimental data were presented as mean ± standard deviation (SD). Significant levels are determined at *p < 0.05, **p < 0.01, ***p < 0.001, and ****p < 0.0001.

We next measured the protein expression levels of the same samples because gene expression does not fully represent subsequent protein expression due to epigenetic changes and functional roles of RNA. We analyzed WISP-1 (a target of the Wnt pathway), as well as BMP-2 (a key osteogenic regulator), under normal and DKK1 conditions to understand the influence of the Wnt pathway on osteogenesis. ELISA assays exhibited that WISP-1 expression doubled in response to PLGA/MgPG5 when compared to PLGA alone (Figure 6C). Additionally, while PLGA/MgPG5 matrices demonstrated similar levels of inhibition in the presence of DKK1, PLGA/MgPG5 displayed strong upregulation of BMP-2 at the protein level under normal media conditions where total protein levels doubled (Figure 6D).

**Discussion**

Bone regeneration is a complex process that requires an ideal combination of matrices, cells, and signaling cues. To achieve complex bone tissue engineering, a proper synthetic matrix must first be constructed with properties that evade the drawbacks associated with current treatment options. This led us to utilize MgPG, consisting of GO along with magnesium and phosphate ions covalently tethered to the GO backbone. With MgPG, we fabricated a bioengineered PLGA/MgPG microsphere-based composite matrix with carefully selected physical properties and desired cellular responses to tune its utility as a bone graft for complex bone regeneration.

Fabricating scaffolds with MgPG at 1 and 5 wt% was a decision made based on previous research in our lab that showed favorable osteogenic properties in similar bone matrices [90]. Such studies demonstrated that among composite PLGA/GO matrices ranging from 1-10 wt%, PLGA/GO5 matrices displayed the highest compressive strength, ALP activity, and alizarin red calcium deposition [90]. Following up on these experiments, through characterization assays, we confirmed the incorporation of our desired material into PLGA microspheres at the desired concentrations of 1 and 5 wt% (Figures 2-3). SEM supports that this integration did not change the morphological characteristics of PLGA microspheres, while EDS supports that the chemical nature of MgPG remained unchanged (Figure 2A). Even within the microspheres, EDS showed that MgPG contained similar quantities of magnesium and phosphorus, corroborated by XPS (Figure 2A, Supplementary Figure 2A). While Arnold et al. has demonstrated that the phosphorus in PGs (such as MgPG) corresponds to phosphates bound to the backbone of GO, this characteristic is upheld in our work by the presence of C-P bonds, verified by high-resolution carbon scans (Supplementary Figure 2B) [61]. Carbon to phosphorus bonds do not account for all the phosphorous in PGs, however, and this is due to polyphosphate formation [97]. The average length of these chains can be calculated utilizing the formula in Supplementary Table 1.

After verifying the successful integration of MgPG into PLGA microspheres at 1 and 5 wt%, all other assays showed characterizations distinct to MgPG except XRD, which did not show the sharp diffraction peak at 11.8° that is characteristic of GO. Instead, our groups previously demonstrated that this typical GO peak shifts toward 26.6° in PGs, which is more characteristic of graphite [61]. The powders of these PGs, including MgPG, display a shifted GO peak at 23° that is less sharp and more broad [61], and this angle overlaps with the broad PLGA 85:15 diffraction peak of 20-23° [98]. The low concentrations of MgPG at 1 and 5 wt% being shielded from the encapsulating PLGA polymer that has an overlapping peak may explain why our XRD results lacked such a distinctive peak (Figure 3C). Additionally, FTIR spectra of all study groups apart from GO demonstrated peaks at ~2800–3000 cm⁻¹ that correspond to C-H sp^2^ and sp^3^ hybridizations (Figure 3D). While it would be expected to see the same peaks for GO, they appear to be absent due to the large and broad overlapping -OH peak (~3350 cm⁻¹). In spite of the overlapping -OH peak, the other two become visible in MgPG-containing groups due to the thermal reduction of MgPG that occurs during its synthesis, which reduces the size of the -OH peak [61].

Regarding matrix properties, PLGA/MgPG composite matrices were superior mechanically with PLGA/MgPG5 being four times stronger than PLGA alone (Figure 4A-C). Although there was a significant increase in the matrices’ UCS, signifying a large increase in strength (e.g. fracture resistance), there was interestingly no change among groups in their compressive moduli, demonstrating that the addition of graphenic materials into PLGA up to 5 wt% did not alter their hardness (e.g. brittleness) characteristic of the encapsulating polymer (Figure 4B-C). The addition of graphenic material to PLGA also increased the hydrophilicity of the composite matrices evidenced by a significant decrease in contact angle measurements (Figure 4D). This increase in hydrophilicity was accompanied by a significant (but not large) increase in swelling of PLGA/GO5 and PLGA/MgPG5 matrices relative to PLGA after 90 days (Figure 4F). This additional water uptake can be beneficial for nutrient delivery to bone defect sites without the fear of swelling too much and causing further damage to the injured tissues. Furthermore, the increased hydrophilicity evidenced by decreased contact angle measurements were also accompanied by an initial significant increase in degradation at day 30 of PLGA/GO5 and PLGA/MgPG5 matrices relative to PLGA alone (Figure 4F-G). These phenomena are expected, as the addition of GO and its further magnesium and phosphate functionalization introduce hydrophilic groups that have been shown to strengthen these properties [99]. Due to the slow degradation rate of PLGA 85:15 (the major component), however, after 90 days, we did not observe a difference in weight loss percentage across groups (Figure 4F). In a study conducted by Perron et al., for example, PLGA 85:15 scaffolds demonstrated only 4% weight loss after 90 days [100]. Similarly for another PLGA 85:15 implant synthesized similarly to our own matrices, Ramchandani et al. exhibited its minimal weight loss (~10%) after the same amount of time (~12.8 weeks); both of which corroborate our observations [101]. These results imply that it may take longer than 90 days to observe the acceleration of degradation rate due to the addition of GO and MgPG, as Ramchandani et al. characterized the degradation of PLGA by a period of stagnation with minimal weight loss followed by a rapid period of weight loss, which left their implants with half of its weight after ~18 weeks and complete degradation after ~26 weeks [101]. While PLGA releases acidic byproducts during its degradation, no change in the pH of the surrounding PBS environment was observed due to the minimal degradation at 90 days (~5%) (Figure 4E). Though the pH of the PBS varied slightly due to its replacement every 2 weeks, the pH of scaffold-containing samples was no different than the PBS-alone samples at every time point (Figure 4E). The functionalization of graphene oxide with basic magnesium and phosphate ions, however, is expected to neutralize the acidic byproduct release by PLGA as other groups have shown [102, 103].

In addition to improving the physical properties of the matrix through functionalization, magnesium and phosphate have also been proven to be great inducerons for the stimulation of bone healing within complex defect sites (such as critical sized defects) via incorporation into synthetic bone grafts [60, 63]. In support of this idea, Burmester et al. demonstrated that osteoblasts cultured in media extracts containing magnesium ions were morphologically larger in diameter and spreading area, which are characteristic of earlier differentiation. Furthermore, they exhibited significantly higher gene expression levels of ALP, BSP, OPN, and RANKL after one week. Even more convincingly, they demonstrated that osteoblasts cultured on pure magnesium samples, when compared to control osteoblasts on tissue culture plates (TCP), promote significantly higher gene expression of ALP, BSP, OCN, OPN, and RANKL [104]. These results provide conclusive data that support magnesium being an induceron, as the metal alone induced and increased osteogenic differentiation. As a major component of bone, phosphate has also been extensively studied (oftentimes alongside calcium) as known inducerons [60, 105, 106].

Corroborating the above findings, our studies also showed that the incorporation of MgPG at 1 and 5 wt% into PLGA matrices increased their capabilities of supporting cell growth/viability, as well as osteogenic potential (Figure 5). Live/Dead and MTS exhibited PLGA/MgPG matrices’ cytocompatibility comparable to that of FDA-cleared PLGA (Figure 5A-B). The GO in MgPG incorporation has been shown by other groups to support cell adhesion and proliferation as well [107, 108]. The increase in ALP activity and calcium deposition from PLGA/MgPG matrices further demonstrated their enhanced osteogenic activity. While the ALP activity in response to PLGA/MgPG1 matrices was not statistically different from PLGA matrices at any time point, PLGA/MgPG5 matrices displayed significantly higher ALP activity than PLGA and PLGA/GO5 matrices at days 10 and 14 (Figure 5C). Similarly, PLGA/MgPG1 matrices led to no statistical difference in calcium deposition by MC3T3 cells up to 28 days relative to PLGA in osteogenic media (Figure 5D). MC3T3 cells seeded on PLGA/MgPG5 matrices, on the other hand, demonstrated significantly higher calcium deposition than PLGA alone on days 14 and 28 (Figure 5D). While PLGA/MgPG5 matrices demonstrated strong mineralization activity under osteogenic media, they also interestingly displayed similar behavior in complete 𝛼MEM without osteogenic factors (ascorbic acid and β-glycerophosphate) (Supplementary Figure 3). These results demonstrate the positive effects of magnesium and phosphate ions on the backbone of GO as inducerons with the capability of inducing osteogenic differentiation in the absence of external factors. GO and MgPG integration into PLGA matrices at 1 wt% may not have been enough to evoke a strong increase in osteogenic potential, but integration at 5 wt% certainly was. Because PLGA/MgPG5 matrices demonstrated significant results in these osteogenic assays while PLGA/MgPG1 matrices did not, we proceeded only with PLGA/MgPG5 as experimental matrices.

Our last studies showed that the Wnt pathway is at least partially responsible for the enhanced osteogenic outcomes of our MgPG studies, validated by an increased gene expression profile of Wnt and osteogenic targets at the gene and protein levels in response to PLGA/MgPG matrices (Figure 6). Under DKK1 conditions, Wnt-related β-catenin was downregulated as well as the WISP-1 target protein for both PLGA and PLGA/MgPG5, demonstrating DKK1’s Wnt pathway specificity. There was still an observed significant increase in β-catenin expression in response to PLGA/MgPG5 matrices, however, perhaps due to the osteoinductive properties of the material, carrying magnesium and phosphate inducerons (Figure 6A). While both the Wnt-pathway and osteogenic markers displayed increased gene expression in response to PLGA/MgPG5, the lower expression levels of the osteogenic protein BMP-2 under DKK1 conditions signify dependence on the Wnt pathway.

An interesting future confirmation assay for the claim that the increased activation of the Wnt pathway by magnesium ions incorporated into the phosphate functionalized GO would be to culture the cells on matrices with and without DKK1, then perform ALP and ARS. This would allow us to study MgPG’s stimulating effects of the Wnt pathway on other osteogenic processes. Additionally, future analysis of the osteogenic gene expression profile in response to PLGA/MgPG matrices under basal media conditions with and without the addition of activators (e.g. Li^+^) or inhibitors may reveal a synergistic effect with the activator or demonstrate a rescue effect where MgPG can bypass inhibitory effects and induce higher gene expression of Wnt markers as we have also observed. A similar pattern was seen in a study in which Wu et al. was able to generate a matrix that was capable of bypassing the inhibitory effects of cardamonin on the Wnt pathway via higher levels of expression of Wnt3a, Frizzled-6 and Axin2 [82]. In our study, we used DKK1 instead of cardamonin, because cardamonin is mostly associated with inhibiting Wnt to combat cancer, which is outside the scope of this study [109]. Furthermore, cardamonin inhibits the pathway at the β-catenin level, whereas DKK1 is a further upstream inhibitor at the LRP level. In our future studies, we can use another Wnt inhibitor (e.g. cardamonin or sclerostin), to see if MgPG can bypass other inhibitors of the Wnt pathway or if its role is by acting more downstream or upstream in the pathway. It would also be intriguing to look at the positive contributions of other pathways that MgPG may affect, as Días-Tocados et al. saw an increase in osteogenic ability of their bone matrices after Mg supplementation, but it was through the notch pathway and not the canonical Wnt [110]. This would be of particular interest to us, because though MgPG5 promoted a significant increase in β-catenin, this increase was not a large one which may imply that there are other pathways involved in the achievement of the enhanced osteogenic ability of MgPG-containing matrices we observed.

Behind these results of our own studies, however, is the utilization of MgPG in PLGA at 1 and 5 wt%, which led to a theoretical amount of magnesium ion presence of 1.0 and 6.7 mM, which is within the range that has been used in other studies. To deliver magnesium ions to cells for bone regeneration, many scientists have included magnesium sulfate (MgSO_4_) into the extract of their media. Burmester et al. did this with osteoblasts in a media solution of 0.813 mM Mg^2+^ and found increased osteoblast diameters, indicating enhanced osteogenic activity. Furthermore, observed increased gene expression of various osteogenic markers in the presence of 0.813 mM Mg^2+^, as well as a further increase in the presence of 0.813 mM Mg^2+^ combined with an osteogenic media [104]. In another study by Hung et al., they utilized a higher concentration of 10 mM Mg^2+^ in basal media and found a large increase in the activation of the osteogenic Wnt pathway in human bone marrow stem cells (hBMSCs) [111]. Other studies such as Yoshizawa et al. look at a range of Mg^2+^ concentrations from 0.8 mM to 100 mM and found the best pro-osteogenic results from 10 mM Mg^2+^ [112]. The highly favorable results found from PLGA/MgPG composite matrices containing up to 6.7 mM Mg^2+^ while other studies have used slightly higher amounts of Mg^2+^ is a result of the additional benefit that comes from the GO and phosphate ions that are also incorporated into PLGA. It may be further beneficial, though, to increase the amount of Mg^2+^ present to be more consistent with the 10 mM Mg^2+^ that other studies have shown to be most favorable, as more magnesium may lead to more Wnt pathway activation and ultimately better bone regeneration efficacy.

One way to increase the amount of magnesium is by manipulating the weight percentages of MgPG incorporated into PLGA. Previous work in our lab showed that there is a tradeoff, though, because although it may be beneficial to add more graphenic powder (in this case MgPG) to obtain more Mg^2+^, increasing the amount of MgPG incorporation too much will also decrease other important properties such as heat-sintering efficiency and mechanical strength. Therefore, it may also be useful to explore how to increase the amount of magnesium on the GO sheets so that 5 wt% incorporation of graphenic materials can still be utilized (as previous work in our lab has shown to be best) while still delivering more magnesium [90]. Of course, the addition of more magnesium to the GO sheets may affect the properties of the matrix in unexpected ways, though, demonstrating the importance for this avenue to be tested. Furthermore, it is important to note that the proposed 6.7 mM concentration of Mg^2+^ is based on theoretical calculations, so it would also be important for future studies to measure the actual amount of Mg^2+^ present within and released from the matrices, then still applying the same logic as above. The goal of this paper was not to optimize the amount of Mg^2+^ released from our matrices to maximize bone regeneration efficacy, but rather, to test and understand the effects of incorporating MgPG into PLGA microspheres for enhanced bone regeneration. Future studies may aim to discover the optimal delivery of Mg^2+^ and PO_4_^3-^ from the novel PLGA/MgPG composite microsphere-based matrices for the best bone regeneration efficacy. One particularly useful test would be atomic absorption spectroscopy to measure the actual amount of magnesium released from our matrices overtime. Knowing this would help to understand the rate of release so that it may be optimized for bone regeneration, as well as the raw amount of MgPG powder to incorporate into PLGA matrices for long-term effects.

For our future studies, it would also be advantageous to repeat them using stem cells (bone marrow stromal/mesenchymal) as opposed to MC3T3 (now that the proof of concept has been shown) for increased translatability. Additionally, moving from *in vitro* to *in vivo* in a rabbit ulnar/radial animal model would also tell a more complete story, as it would show MgPG’s ability to actually regenerate bone tissue rather than its sole potential to do so via osteogenic assays (Supplementary Figure 4). Previously, a similar *in vivo* study was conducted by our groups involving 3D-printed matrices (composed of 10% PLGA and 90% Calcium Phosphate Graphene (CaPG)) within mouse calvarial defects [48]. Given the extremely high porosity (~95%) and structural differences of the PLGA/CaPG matrices, the PLGA/CaPG matrices were about eight times weaker than our PLGA/MgPG5 matrices and more suitable for a non-weight bearing defect area (Figure 4A-B) [48]. The superior strength and slow degradation (> 3 months) of PLGA/MgPG5 makes it a great scaffold candidate for the previously mentioned rabbit ulnar/radial model defect which is weight bearing. This defect model has been established in our lab previously and continues for 3 months [33]. Though there was not much degradation observed after 3 months in PBS (Figure 4F), the dynamic environment as well as the presence of macrophages and enzymes *in vivo* will modulate potential acidity and matrix degradation to allow for the integration of new bone [113]. Other interesting factors to consider *in vivo* would be if MgPG displays any sex or age differences in the bone regeneration outcomes of their hosts. Many groups have shown sex-dependent differences in fracture healing such as Andrew et al. who demonstrated that female mice have more estrogen receptor 2 on their skeletal stem cells, leaving them dependent upon estradiol during fracture healing while male mice were unresponsive to it [114]. Additionally, Rude et al. showed that insufficient dietary magnesium intake in young and mature mice caused hypomagnesemia and led to impaired bone growth [115]. Other studies have shown a connection between hypomagnesemia and osteoporosis, a bone disease that mostly affects older women, which makes the exploration of MgPG on sex and age differences *in vivo* more compelling [116-119].

The ultimate outlook for MgPG is for it to be used to regenerate bone within critical sized defects in humans. A current limitation is that, before this can be achieved, GO will need to undergo clinical trials to become FDA-cleared for tissue engineering, as did PLGA [120]. To this end, GO is currently undergoing a phase one clinical trial to prove its biosafety [121]. Although the GO is being inhaled rather than implanted, this study may lay the groundwork for GO becoming a powerful FDA-cleared material for uses such as bone regenerative engineering. With this hope, further in-human studies are needed, such as exploring different doses, durations, and potential susceptibility in larger cohorts as well as further *in vitro/in vivo* assays using GO before it can be translated into practice. Such tests will help develop innovative strategies for bone regeneration by providing the framework for utilizing graphenic materials such as MgPG to enhance tissue engineering. Once there, MgPG matrices have many unique advantages such as easily tunable properties, enhanced mechanical strength, and favorable cell interaction and osteogenic properties. Understanding the mechanisms by which MgPG matrices enhance bone regeneration properties is essential for the development of effective therapies in the field of orthopaedic surgery and tissue engineering. Future efforts may seek to analyze and manipulate other properties of the PLGA/MgPG matrix to bring forth specific characteristics such as those in a spatial manner. For example, a MgPG bone matrix could be synthesized with different pore sizes throughout via varying sizes of microspheres. Large microspheres have already been shown to create larger pore sizes and high cellular interactions, though these characteristics leave the matrix weaker [122, 123]. On the other hand, smaller microspheres have demonstrated the generation of higher mechanical properties with smaller pore sizes, though it disrupts cellular infiltration [122, 123]. Another avenue may be to utilize microspheres with different concentrations of MgPG throughout the matrix for the healing of different tissue types such as the bone and cartilage at the osteochondral interface [124]. To achieve optimization of these parameters with specificity, it would be essential to have precise control over fabrication methods to ensure microsphere-loading and size consistency between matrices. Such regulation could be achieved via a microfluidic device for the synthesis of uniform microspheres, though the process may not be very time effective. Utilizing 3D-printing would overcome the limitation of scalability, as it would allow the precise, accurate, and rapid fabrication of various composite MgPG matrix designs, leading to improved clinical treatments for bone-related disorders for orthopaedic surgeons and their many patients.

**Conclusion**

In conclusion, this paper has comprehensively investigated the physicochemical properties, cytocompatibility, and osteogenic potential of an MgPG bone scaffold while also elucidating a potential underlying mechanism. Specifically, our groups have demonstrated the successful fabrication of composite PLGA and MgPG microsphere-based matrices for bone regenerative engineering. The incorporation of MgPG at 1 and 5 weight percentages significantly enhanced the mechanical strength of the encapsulating PLGA. Additionally, the release of Mg^2+^ and PO_4_^3-^ ions from PLGA/MgPG composite matrices promoted significantly higher ALP and calcium deposition, revealing their enhanced osteogenic properties. Furthermore, gene and protein expression assays exhibited a significant increase in Wnt pathway markers/targets (β-catenin and WISP-1) as well as osteogenic markers (Col1a1, OCN, BSP, and Sp7) with and without a Wnt-pathway-specific inhibitor (BMP-2). These results show that the Wnt pathway activation in response to PLGA/MgPG composite matrices is a contributor to the observed enhanced osteogenesis. With further study, particularly analyzing *in vivo* effects, PLGA/MgPG matrices may prove to be great candidates for the healing of complex bone defects. The outcomes of this research have the potential to significantly impact the development of advanced therapies for bone-related disorders that can be used by orthopedic doctors for their patients.

**Materials and Methods**

**Synthesis of Graphene Oxide (GO)**

GO was made using a modified Hummer method. A 4 L flask was chilled over ice, followed by adding 250 mL of concentrated sulfuric acid (Fisher). After adding 10 g graphite flakes (325 mesh, 99.8% metal basis; Alfa Aesar) to the flask, 20 g of KMnO_4_ (99.0% crystalline, Alfa Aesar) was added slowly over 20 - 30 minutes with constant stirring. The reaction mixture was warmed to room temperature and stirred for 2 hours. Then, the reaction mixture was warmed to 35^o^C to stir for two additional hours. After heating, the mixture was removed from heat, and the reaction was quenched by rapidly adding 1400 mL deionized (DI) water, 20 mL 30% H_2_O_2_ (VWR), and 450 mL more DI water. The resultant mixture was left to stir overnight. The mixture was then vacuum filtered, and the isolated GO was loaded into 3500 molecular weight cutoff dialysis tubing dialyzing (SnakeSkin^TM^ dialysis tubing; Thermo Scientific) against water for seven days with deionized (DI) water changed twice a day. The GO was frozen at -80^o^C and lyophilized for 72 hours until fully dried [61].

**Synthesis of Magnesium Phosphate Graphene (MgPG)**

Five hundred milligrams of synthesized GO, 500 mg magnesium bromide diethyl etherate (Alfa Aesar), and 500 mL of triethyl phosphite (Sigma Aldrich) were added to a flame-dried 3-neck round bottom flask under N_2_. This mixture was sonicated for one hour. Following sonication, 2.5 g of MgBr (Alfa Aesar) was added to the reaction under N_2_, and the mixture was sonicated for an additional 30 minutes. The reaction mixture was refluxed at 156^o^C under N_2_ for 72 hours with vigorous stirring. The MgPG was isolated through vacuum filtration and washed by centrifugation at 3600 g for 5 minutes, twice with acetone, once with DI water, once with ethanol, and finally twice more with acetone. The MgPG was then dried under a vacuum for 48 hours [61].

**Microsphere/Matrix Fabrication**

Poly (DL-lactide-*co*-glycolide) (PLGA 85:15 lactide to glycolide ratio, Mw = 152 kDa, IV = 076 - 0.85 dL/g) was obtained from Evonik Corporation (Birmingham, AL). Polyvinyl alcohol (PVA, 88 - 90% hydrolyzed, average Mw 30,000 - 70,000) was obtained from Sigma-Aldrich (St. Louis, MO). Methylene chloride (dichloromethane, DCM) was purchased from Fisher Scientific (Pittsburgh, PA). An oil-in-water emulsion solvent method was used to prepare PLGA, PLGA/GO, and PLGA/MgPG microspheres. 4000 mg of PLGA was dissolved in 20 mL DCM to yield pure PLGA microspheres (a ratio of 200:1 PLGA (mg):DCM(mL)). The composite microspheres were prepared by adding MgPG and GO powder to PLGA at 1 and 5 wt% (40 and 200 mg of powder, respectively, per 4000 mg of total weight, e.g. PLGA/MgPG1, PLGA/MgPG5) into a vial with 20 mL of DCM. Following complete dissolution and dispersion via shaking, the mixture was vortexed until it formed a homogeneous suspension. Under a stirring speed of 300 rpm, the dissolved polymer (oil phase) was added via pouring with a thin stream into a 1% (w/v) PVA solution (water phase) and allowed to stir overnight at room temperature for complete evaporation of DCM. The following day, the microspheres were washed with deionized water, collected via vacuum filtration, frozen at -80°C overnight, and then lyophilized for 48 hours.

To create 3D porous structures, the microspheres were subsequently sieved to select for microspheres within the size range of 300 - 600 μm. They were then packed into stainless steel cylindrical molds and heat-sintered for 80 minutes at 80°C.

Mechanical and degradation tests were performed using 5*5 mm cylindrical matrices. All other *in vitro* studies were conducted on cylindrical matrices with a diameter of 7 mm and a height of 1 mm unless otherwise stated.

All *in vitro* studies were conducted on cylindrical matrices with a diameter of 7 mm and a height of 1mm.

**Micro Computed Tomography (µCT)**

Samples were imaged on a Scanco µCT40 specimen scanner.

**Scanning Electron Microscopy (SEM) & Energy Dispersive X-Ray Spectroscopy (EDS)**

Imaging of the microspheres and microsphere-based matrices was performed by SEM. Samples were prepared by mounting them, then gold-palladium sputter coating them to be imaged by an FEI Nova NanoSEM 450 with a working distance of 5 mm and an acceleration voltage of 18 kV.

Elemental analysis of the microsphere interior was conducted by slicing microspheres in half with a sharp scalpel blade on the mounting stand before imaging. Subsequently, EDS was completed with an Oxford AZtecEnergy Microanalysis System and X-Max 80 Silicon Drift Detector.

**Thermogravimetric Analysis (TGA)**

The incorporation of MgPG within the PLGA matrices was evaluated using a TGA (TA Instruments TGA Q500). Using a ramp rate of 10°C per min^-1^, TGA was performed under nitrogen at a temperature range from room temperature to 800°C.

**X-Ray Diffraction (XRD)**

XRD was performed on microspheres using a Bruker D2 Phaser. Samples were measured over a range of 0 to 75 2θ. Spectra were smoothed using Microsoft Excel (Period = 50)

**Fourier Transform Infrared (FTIR) Spectroscopy**

Spectra were collected using a PerkinElmer Frontier FT-IR Spectrometer with an attenuated total reflectance (ATR) attachment containing a germanium crystal. The data was recorded as percent transmittance from 4000 to 700 cm^–1^ with a 4 cm^–1^ resolution and smoothed with a boxcar of 10 and then offset for clarity. Spectrum software (Spectrum version 10, PerkinElmer) was utilized for ATR and baseline corrections of raw spectra.

**Mechanical Strength Testing**

An Instron 5544 mechanical tester with a 2 kN load cell was used to evaluate the mechanical properties of the matrices. A uniaxial compressive loading was applied to matrices (n = 6 per group) with a crosshead speed of 2 mm/min and a strain limit of 70%. Compressive strength values were recorded as the maximum load values between the ranges of 0 and 50% strain, inclusive. Compressive moduli were calculated at the onset of the linear region of the stress–strain curve.

**Wettability Assessment**

Wettability assessment included the measurement of a water-in-air contact angle. Samples were prepared by repeating the procedure for making microspheres, but instead of pouring the polymer/DCM mixture into PVA, it was instead pipetted onto and spread flat on glass slides. The slides were then placed in a hot room (37°C) for three days for the DCM to evaporate completely, leaving only the material on the slide. To run the test, a drop of 5 µL of deionized water was automatically placed onto samples using a Dataphysics OCA20 contact angle analyzer and the sessile drop method at room temperature. Images of water absorption were captured with a high-speed framing camera, and measurements were performed through ImageJ software Version 1.53c to record the contact angle of the water drop on the sample.

**Degradation, Swelling, & pH**

Over a period of four months, the percentage of weight loss and swelling of the study groups was measured to assess the degradation behavior of the matrices. Initial matrix weights (n = 6 per group and time point) were recorded. Afterward, matrices were sustained at 37°C under gentle agitation in a 15 mL solution of 1x PBS, which was replaced every two weeks to prevent oversaturation. Further assessments were conducted on the matrices on days 7, 14, 21, 30, 60, and 90.

The pH was measured by inserting a pH probe (Fisherbrand Accumic) into the matrix-containing PBS stocks and reading the measurement. The swelling behavior of the matrices was evaluated by positioning the collected samples under a gentle air flow for a few seconds to blow out extra PBS from the matrices. The samples were then weighed to collect the wet weights. The matrices were subsequently freeze-dried for 48 hours, then weighed to record their dry weights.

Based on the following equation, the percentage of swelling was calculated:

Swelling Rate = (W_w_ – W_o_) / W_o_ * 100%

*Ww* represents the wet weight of the matrix, and *Wo* represents the initial weight of the matrix.

Based on the next equation, the percentage of weight loss was calculated:

Weight Loss = (W_o_ – W_d_) / W_o_ * 100%

*Wd* represents the dry weight of the matrix, and *Wo* represents the initial weight of the matrix.

**Matrix Sterilization**

Matrices were soaked in 70% ethanol for one hour, after which, the matrices were washed two times with PBS. Lastly, the matrices were radiated with UV for 1 hour on each side.

**Cell Culture**

Mouse pre-osteoblast cells (MC3T3-E1 Clone 4 cells) were obtained from *ATCC* (Manassas, VA). MC3T3 cells were grown on a tissue culture flask in a modified Minimum Essential Medium (𝛼MEM) basal medium containing 1% penicillin/streptomycin and 10% fetal bovine serum (FBS) (hereby denoted as “growth media”) and maintained in a humidified incubator kept at 37°C and 5% CO_2_. The medium was changed every three days. At 80% confluence, cells were detached from the culture flasks using TrypLE Express (without phenol red), collected, centrifuged at 200 xg for 10 minutes with the addition of 𝛼MEM to deactivate the trypsin, and then seeded onto sterile matrices placed in 24-well plates with ultra-low attachment (Corning) so the cells do not flow out and preferentially bind to TCP. For all studies, matrices were seeded with 20,000 cells (unless otherwise stated) in a 10 μL droplet of media. The matrices were then incubated for one hour to allow the cells to adhere to the matrices, then 1 mL of growth media was added to the wells. Media was changed every three days.

**Cell Viability Assay**

Cell viability was determined on days 1, 3, 7, and 14 of the growth cycle using the LIVE/DEAD Cell Imaging Kit according to the manufacturer’s instructions (Invitrogen; Carlsbad, CA). At each time point, the matrices (n = 6 per group) were transferred to a new 48 well plate, rinsed twice with Dulbecco's Phosphate-Buffered Saline (DPBS), then incubated in a staining solution for 15 minutes. A confocal microscope (Zeiss LSM Confocor2) at a magnification of 10x was used for imaging.

**Cytocompatibility and Proliferation**

The CellTiter 96 Aqueous One Solution Cell Proliferation Assay system (Promega) was used for cell proliferation assays on days 1, 3, 7, and 14 After transferring the matrices into new wells (n = 6 per group), they were washed once with DPBS and incubated for 2 hours at 37°C with 1:5 dye to media MTS solution (1 μL MTS reagent per 4 μL growth medium). Using a plate reader (BioTek, Synergy H1, Winooski, VT), the absorbance values of the matrices were read at 490 nm. MTS solution was added to empty wells to be used as a blank, which was deducted from the absorbance values of the cell-seeded matrices to analyze the effect of the matrix and cells alone. The analyzed data was normalized to the day 1 (D1) value for each group.

**Alkaline Phosphatase (ALP) Activity**

Matrices were sterilized and seeded with cells as described in the previous section. The cell-seeded matrices were maintained in growth media for 3 days and then switched to osteogenic differentiation media, with replacement every 3 days. The osteogenic differentiation media consisted of growth media (𝛼MEM basal medium containing 1% penicillin/streptomycin and 10% FBS) supplemented with 3 mM β-glycerophosphate (Sigma Aldrich) and 10 μg/mL L-Ascorbic Acid (Fisher Scientific) (hereby denoted as “osteogenic media”). Alkaline phosphatase (ALP) activity was measured for all groups at 3, 5, 7, 10, and 14 days post-osteogenic media addition (n=6 per group per time point). At each time point, the cell-seeded matrices were collected, washed once with DPBS, then placed (dry) into a 48 well plate. Upon the completion of all time points, 200 μL of 1% Triton were added to the matrices, and they underwent three cycles of freeze thawing to lyse the cells. ALP activity was measured via Alkaline Phosphatase Substrate Kit (BioRad; Hercules, CA) following the manufacturer instructions. Absorbance at 405 nm was measured using a Varioskan Flash spectral scanning multimode reader after 90 minutes of incubation at 37℃. ALP solution was added to empty wells to be used as a blank, which was deducted from the absorbance values of the cell-seeded matrices to analyze the effect of the matrix and cells alone. The ALP activity data was normalized to the amount of protein present in the cell lysates at each timepoint by following the BCA kit’s manufacture instructions (Thermo Scientific). The data was further standardized to day 3 values for each group.

**Alizarin Red Staining (ARS)**

Matrices were sterilized and seeded with cells as described in the previous section. The cell-seeded matrices for one set were maintained in growth media for 3 days and then either switched to osteogenic media or continued in growth media, with replacement every 3 days. The other set went through the same process but was never exposed to osteogenic media. The growth media was 𝛼MEM basal medium (Invitrogen) alone. Bone mineralization was measured via Alizarin Red staining (ARS) for all groups at 14, 21, and 28 days post-osteogenic media addition (n=6 per group per time point). At each time point, the cell-seeded matrices were collected, washed once with PBS, then placed (dry) into a 48 well plate. Upon the completion of all time points, the samples were transferred to new well plates, rinsed with DI water and fixed in 70% ethanol for 1 hour at 4°C. The samples were then air-dried for 5 – 10 mins, washed once with DI water, and incubated in the Alizarin Red S dye for 20 minutes at room temperature in the dark. The stained samples were subsequently washed with DI water several times. Quantification was performed by extracting the Alizarin Red S stain using a 10% CPC solution (both purchased from Millipore Sigma; St. Louis, MO). After 30 minutes of incubation at room temperature, the absorbance of the resulting solution was measured at 562 nm using a TECAN plate reader. ALP solution was added to empty wells to be used as a blank, which was deducted from the absorbance values of the cell-seeded matrices to analyze the effect of the matrix and cells alone. The ARS data was normalized to the amount of protein present in the cell lysates of the matrices (n = 3) at each timepoint by following the BCA kit’s manufacture instructions (Thermo Scientific). The data was further standardized to day 14 values for each group.

**Real Time Reverse Transcription Quantitative Polymerase Chain Reaction (RT-qPCR)**

MC3T3 cells were cultured and grown as previously described, then serum starved overnight upon reaching 80% confluence. The following day, cells were detached from the culture flasks using TrypLE Express (without phenol red), collected, centrifuged at 200 xg for 10 minutes with the addition of media to deactivate the trypsin, and then seeded onto sterile matrices placed in 24-well plates with ultra-low attachment (Corning) for preferential binding to the matrix rather than the TCP. The matrices (n = 6) were then seeded with 50,000 cells each in a 10 μL droplet of media (for a total of 300,000 cells seeded per group; this was done three times for n = 3 biological replicates). The matrices were then incubated for one hour to allow the cells to adhere to the matrices. After attachment, the samples were incubated in 1 mL of either the previously described media or media treated with either 100 ng/mL of PBS (control) or DKK1 (inhibitor) and replaced every 48hrs.

After 7 days, the matrices were washed two times with PBS before preparing and pooling the cells lysates from all samples for each group using the trizol/chloroform method. Centrifugation at 13,000 xg for 15 minutes at 4°C induced phase separation, and the aqueous phase was extracted for RNA isolation, while the protein pellet was frozen in liquid nitrogen and stored for ELISA.

Total RNA was isolated from the aqueous phase of the phase-separated cell lysate using the RNeasy Mini Kit (Qiagen, Hilden, Germany) according to the manufacturer’s instructions. Three micrograms of mRNA was then used to synthesize complementary DNA with the RNA to cDNA EcoDry Premix (Clontech, Mountain View, CA). RT-qPCR was run in a light cycler instrument (Bio-Rad iCycler iQ System, Hercules, CA) on 20 μL of mastermix for each group/primer/condition consisting of 10 μL of cDNA, 9 μL of SuperMix Premix (Biorad, Hercules, CA), and 1 μL of Taqman primers (Applied Biosystems, Guilford, CT) shown in Table 1. The relative fold expression of the genes of interest were normalized to the housekeeping gene, GAPDH, and calculated using the ΔΔCT method.

**Table 1: Genes/Primers Used in qPCR to Study the Wnt Pathway and Osteogenic Markers**

| Gene Name | Primer Sequences |
| --- | --- |
| Ctnnb1 (β-catenin) | 5’ – GTTCGCCTTCATTATGGACTGCC – 3’  5’ – ATAGCACCCTGTTCCCGCAAAG – 3’ |
| SP7 | 5' - TTTACTCCCTTGTCCTCACC - 3'  5' - GGAAGACGTAGAGTTAGCTG - 3' |
| COL1A1 | 5' - GCTGGTCCTGTTGTATGTAGC - 3'  5' - CCAGCACCATATGGTAGGGGCACAT - 3' |
| BSP | 5' – CTGCTTCCTCACTCCAGGAC - 3'  5' – ATTGAGAAAGCACAGGCCAT - 3' |
| BGLAP | 5' – ACCAGTATGGCTTGAAGACCGC - 3'  5' – TTTTGGAGCTGCTGTGACATCC - 3' |
| GAPDH | 5’ – CATCACTGCCACCCAGAAGACTG – 3’  5’ – TGCCAGTGAGCTTCCCGTTCAG – 3’ |

**Enzyme-Linked Immunosorbent Assay (ELISA)**

The frozen pellets from the previous RT-qPCR section were thawed on ice, then centrifuged at 21,000 xg for 15 minutes at 4°C to pellet the protein. After discarding the supernatant, the protein pellet was resuspended in PBS, then sonicated to aid in dissolution. Total protein was determined by means of the BCA assay according to the manufacturer’s instructions (Thermo Scientific). The same amount of total protein was subsequently added into the ELISA wells according to each kit’s specific manuals (R&D Systems; BMP-2: DBP200, and WISP-1: MWSP10) followed by the manufacturer’s instructions to measure the amounts of Wnt3a and WISP-1 protein expression levels of the samples (n=4).

**Statistical Analysis**

GraphPad Prism 10 (GraphPad Software; San Diego, CA) was used for statistical analysis and graph design. Quantitative experimental data were presented as mean ± standard deviation (SD). Statistical analysis of the data was performed using a one- or two-way analysis of variance (ANOVA), and comparison between two means was determined by a Šidák test. Significant levels are determined at *p < 0.05, **p < 0.01, ***p < 0.001, and ****p < 0.0001.

**Acknowledgments/Funding**

This work was gratefully supported by the NIH T32 (AR079114) and NSF EFRI-BioFlex (1332329) grants. Additionally, figures were created with BioRender.com.

References

1.	Riggs BL, Melton Iii L. The worldwide problem of osteoporosis: insights afforded by epidemiology. *Bone* 1995;17:S505-S511.

2.	Roddy E, DeBaun MR, Daoud-Gray A, Yang YP, Gardner MJ. Treatment of critical-sized bone defects: clinical and tissue engineering perspectives. *Eur J Orthop Surg Traumatol* 2018;28:351-362.

3.	Nauth A, Schemitsch E, Norris B, Nollin Z, Watson JT. Critical-Size Bone Defects: Is There a Consensus for Diagnosis and Treatment? *J Orthop Trauma* 2018;32 Suppl 1:S7-S11.

4.	Keating JF, Simpson AH, Robinson CM. The management of fractures with bone loss. *J Bone Joint Surg Br* 2005;87:142-50.

5.	Hosseini FS, Soleimanifar F, Ardeshirylajimi A, Vakilian S, Mossahebi-Mohammadi M, Enderami SE, Khojasteh A, Zare Karizi S. In vitro osteogenic differentiation of stem cells with different sources on composite scaffold containing natural bioceramic and polycaprolactone. *Artif Cells Nanomed Biotechnol* 2019;47:300-307.

6.	Baldwin P, Li DJ, Auston DA, Mir HS, Yoon RS, Koval KJ. Autograft, Allograft, and Bone Graft Substitutes: Clinical Evidence and Indications for Use in the Setting of Orthopaedic Trauma Surgery. *J Orthop Trauma* 2019;33:203-213.

7.	Laurencin CT, Hosseini FS, Daneshmandi L. Correction: Regenerative Engineering: From Convergence to Consilience. *Regenerative Engineering and Translational Medicine* 2024.

8.	Laurencin CT, Khan Y. Regenerative engineering. *Sci Transl Med* 2012;4:160ed9.

9.	Laurencin CT, Nair LS. The Quest toward limb regeneration: a regenerative engineering approach. *Regen Biomater* 2016;3:123-5.

10.	Abedini AA, Hosseini F, Laurencin CT. Regenerative Engineering of a Limb: From Amputation to Regeneration. *Regenerative Engineering and Translational Medicine* 2023;10:461-479.

11.	Barui S, Ghosh D, Laurencin CT. Osteochondral regenerative engineering: challenges, state-of-the-art and translational perspectives. *Regen Biomater* 2023;10:rbac109.

12.	Esdaille CJ, Washington KS, Laurencin CT. Regenerative engineering: a review of recent advances and future directions. *Regen Med* 2021;16:495-512.

13.	Otsuka T, Phan AQ, Laurencin CT, Esko JD, Bryant SV, Gardiner DM. Identification of Heparan-Sulfate Rich Cells in the Loose Connective Tissues of the Axolotl (Ambystoma mexicanum) with the Potential to Mediate Growth Factor Signaling during Regeneration. *Regen Eng Transl Med* 2020;6:7-17.

14.	Otsuka T, Kan HM, Mengsteab PY, Tyson B, Laurencin CT. Fibroblast growth factor 8b (FGF-8b) enhances myogenesis and inhibits adipogenesis in rotator cuff muscle cell populations in vitro. *Proc Natl Acad Sci U S A* 2024;121:e2314585121.

15.	Gonsalves KE, Halberstadt CR, Laurencin CT, Nair LS. *Biomedical nanostructures*. John Wiley & Sons, 2007.

16.	Vinikoor T, Dzidotor GK, Le TT, Liu Y, Kan HM, Barui S, Chorsi MT, Curry EJ, Reinhardt E, Wang H, Singh P, Merriman MA, D'Orio E, Park J, Xiao S, Chapman JH, Lin F, Truong CS, Prasadh S, Chuba L, Killoh S, Lee SW, Wu Q, Chidambaram RM, Lo KWH, Laurencin CT, Nguyen TD. Injectable and biodegradable piezoelectric hydrogel for osteoarthritis treatment. *Nat Commun* 2023;14:6257.

17.	Hinton JL, Jr., Warejcka DJ, Mei Y, McLendon RE, Laurencin C, Lucas PA, Robinson JS, Jr. Inhibition of epidural scar formation after lumbar laminectomy in the rat. *Spine (Phila Pa 1976)* 1995;20:564-70; discussion 579-80.

18.	Ogueri KS, Escobar Ivirico JL, Li Z, Blumenfield RH, Allcock HR, Laurencin CT. Synthesis, Physicochemical Analysis, and Side Group Optimization of Degradable Dipeptide-Based Polyphosphazenes as Potential Regenerative Biomaterials. *ACS Appl Polym Mater* 2019;1:1568-1578.

19.	Nair LS, Laurencin CT. Nanofibers and nanoparticles for orthopaedic surgery applications. *J Bone Joint Surg Am* 2008;90 Suppl 1:128-31.

20.	Shah S, Esdaille CJ, Bhattacharjee M, Kan HM, Laurencin CT. The synthetic artificial stem cell (SASC): Shifting the paradigm of cell therapy in regenerative engineering. *Proc Natl Acad Sci U S A* 2022;119.

21.	Shah S, Ghosh D, Otsuka T, Laurencin CT. Classes of Stem Cells: From Biology to Engineering. *Regen Eng Transl Med* 2024;10:309-322.

22.	Mirdamadi ES, Attari S, Laurencin CT. Standardizing clinical trials for synthetic matrices in rotator cuff tears. *Nature Reviews Bioengineering* 2025.

23.	Barajaa MA, Ghosh D, Laurencin CT. Decellularized Extracellular Matrix-Derived Hydrogels: a Powerful Class of Biomaterials for Skeletal Muscle Regenerative Engineering Applications. *Regenerative Engineering and Translational Medicine* 2023;11:39-63.

24.	Barajaa MA, Otsuka T, Ghosh D, Kan HM, Laurencin CT. Development of porcine skeletal muscle extracellular matrix-derived hydrogels with improved properties and low immunogenicity. *Proc Natl Acad Sci U S A* 2024;121:e2322822121.

25.	Tang X, Daneshmandi L, Awale G, Nair LS, Laurencin CT. Skeletal Muscle Regenerative Engineering. *Regen Eng Transl Med* 2019;5:233-251.

26.	Esdaille CJ, Ude CC, Laurencin CT. Regenerative Engineering Animal Models for Knee Osteoarthritis. *Regen Eng Transl Med* 2022;8:284-297.

27.	Dzidotor GK, Moorhead JB, Jr., Ude CC, Ogueri KS, Ghosh D, Laurencin CT. Functions and Effectiveness of Unloader, Patellofemoral, and Knee Sleeve Orthoses: A Review. *Regen Eng Transl Med* 2024;10:147-171.

28.	Bhattacharjee M, Escobar Ivirico JL, Kan HM, Shah S, Otsuka T, Bordett R, Barajaa M, Nagiah N, Pandey R, Nair LS, Laurencin CT. Injectable amnion hydrogel-mediated delivery of adipose-derived stem cells for osteoarthritis treatment. *Proc Natl Acad Sci U S A* 2022;119.

29.	Chapman JH, Ghosh D, Attari S, Ude CC, Laurencin CT. Animal Models of Osteoarthritis: Updated Models and Outcome Measures 2016-2023. *Regen Eng Transl Med* 2024;10:127-146.

30.	Ude CC, Shah S, Ogueri KS, Nair LS, Laurencin CT. Stromal Vascular Fraction for Osteoarthritis of the Knee Regenerative Engineering. *Regen Eng Transl Med* 2022;8:210-224.

31.	Merriman Jr MA, Chapman JH, Whitfield T, Hosseini F, Ghosh D, Laurencin CT. Fat Expansion Not Fat Infiltration of Muscle Post Rotator Cuff Tendon Tears of the Shoulder: Regenerative Engineering Implications. *Regenerative Engineering and Translational Medicine* 2023;11:1-14.

32.	Laurencin CT, Jiang T. *Bone Graft Substitutes and Bone Regenerative Engineering*. ASTM International, 2014.

33.	Awale GM, Barajaa MA, Kan HM, Seyedsalehi A, Nam GH, Hosseini FS, Ude CC, Schmidt TA, Lo KW, Laurencin CT. Regenerative engineering of long bones using the small molecule forskolin. *Proc Natl Acad Sci U S A* 2023;120:e2219756120.

34.	Hosseini FS, Laurencin CT. Advanced Electrospun Nanofibrous Stem Cell Niche for Bone Regenerative Engineering. *Regenerative Engineering and Translational Medicine* 2022;9:165-180.

35.	Yu X, Botchwey EA, Levine EM, Pollack SR, Laurencin CT. Bioreactor-based bone tissue engineering: the influence of dynamic flow on osteoblast phenotypic expression and matrix mineralization. *Proc Natl Acad Sci U S A* 2004;101:11203-8.

36.	Wang J, Valmikinathan CM, Liu W, Laurencin CT, Yu X. Spiral-structured, nanofibrous, 3D scaffolds for bone tissue engineering. *J Biomed Mater Res A* 2010;93:753-62.

37.	Abdel-Fattah WI, Jiang T, El-Bassyouni GE-T, Laurencin CT. Synthesis, characterization of chitosans and fabrication of sintered chitosan microsphere matrices for bone tissue engineering. *Acta Biomaterialia* 2007;3:503-514.

38.	Ambrosio AM, Sahota JS, Khan Y, Laurencin CT. A novel amorphous calcium phosphate polymer ceramic for bone repair: I. Synthesis and characterization. *J Biomed Mater Res* 2001;58:295-301.

39.	Borden M, Attawia M, Khan Y, El-Amin SF, Laurencin CT. Tissue-engineered bone formation in vivo using a novel sintered polymeric microsphere matrix. *J Bone Joint Surg Br* 2004;86:1200-8.

40.	Amini AR, Laurencin CT, Nukavarapu SP. Bone tissue engineering: recent advances and challenges. *Crit Rev Biomed Eng* 2012;40:363-408.

41.	Hosseini F, Nair L, Laurencin C. Inductive Materials for Regenerative Engineering. *Journal of Dental Research* 2021:00220345211010436.

42.	Nelson C, Khan Y, Laurencin CT. Nanofiber/Microsphere Hybrid Matrices In Vivo for Bone Regenerative Engineering: A Preliminary Report. *Regen Eng Transl Med* 2018;4:133-141.

43.	Meyer JC, Geim AK, Katsnelson MI, Novoselov KS, Booth TJ, Roth S. The structure of suspended graphene sheets. *Nature* 2007;446:60-3.

44.	Balandin AA, Ghosh S, Bao W, Calizo I, Teweldebrhan D, Miao F, Lau CN. Superior thermal conductivity of single-layer graphene. *Nano Lett* 2008;8:902-7.

45.	Stoller MD, Park S, Zhu Y, An J, Ruoff RS. Graphene-based ultracapacitors. *Nano Lett* 2008;8:3498-502.

46.	Lee C, Wei X, Kysar JW, Hone J. Measurement of the elastic properties and intrinsic strength of monolayer graphene. *Science* 2008;321:385-8.

47.	Daneshmandi L, Barajaa M, Tahmasbi Rad A, Sydlik SA, Laurencin CT. Graphene-Based Biomaterials for Bone Regenerative Engineering: A Comprehensive Review of the Field and Considerations Regarding Biocompatibility and Biodegradation. *Adv Healthc Mater* 2021;10:e2001414.

48.	Daneshmandi L, Holt BD, Arnold AM, Laurencin CT, Sydlik SA. Ultra-low binder content 3D printed calcium phosphate graphene scaffolds as resorbable, osteoinductive matrices that support bone formation in vivo. *Sci Rep* 2022;12:6960.

49.	Ding L, Xu T, Zhang J, Ji J, Song Z, Zhang Y, Xu Y, Liu T, Liu Y, Zhang Z, Gong W, Wang Y, Shi Z, Ma R, Geng J, Ngo HT, Geng F, Liu Z. Covalently bridging graphene edges for improving mechanical and electrical properties of fibers. *Nat Commun* 2024;15:4880.

50.	Hosseini FS, Abedini AA, Chen F, Whitfield T, Ude CC, Laurencin CT. Oxygen-Generating Biomaterials for Translational Bone Regenerative Engineering. *ACS Appl Mater Interfaces* 2023.

51.	Hosseini FS, Laurencin CT. Advanced graphene ceramics and their future in bone regenerative engineering. *International Journal of Applied Ceramic Technology* 2022;19:893-905.

52.	Qi Y, Chen W, Liu F, Liu J, Zhang T, Chen W. Aggregation morphology is a key factor determining protein adsorption on graphene oxide and reduced graphene oxide nanomaterials. *Environmental Science: Nano* 2019;6:1303-1309.

53.	Lee WC, Loh KP, Lim CT. When stem cells meet graphene: Opportunities and challenges in regenerative medicine. *Biomaterials* 2018;155:236-250.

54.	Suk JW, Piner RD, An J, Ruoff RS. Mechanical properties of monolayer graphene oxide. *ACS Nano* 2010;4:6557-64.

55.	Nayak TR, Andersen H, Makam VS, Khaw C, Bae S, Xu X, Ee P-LR, Ahn J-H, Hong BH, Pastorin G. Graphene for controlled and accelerated osteogenic differentiation of human mesenchymal stem cells. *ACS nano* 2011;5:4670-4678.

56.	Wu X, Zheng S, Ye Y, Wu Y, Lin K, Su J. Enhanced osteogenic differentiation and bone regeneration of poly (lactic-co-glycolic acid) by graphene via activation of PI3K/Akt/GSK-3β/β-catenin signal circuit. *Biomaterials science* 2018;6:1147-1158.

57.	Peng S, Feng P, Wu P, Huang W, Yang Y, Guo W, Gao C, Shuai C. Graphene oxide as an interface phase between polyetheretherketone and hydroxyapatite for tissue engineering scaffolds. *Sci Rep* 2017;7:46604.

58.	Gillman CE, Jayasuriya AC. FDA-approved bone grafts and bone graft substitute devices in bone regeneration. *Mater Sci Eng C Mater Biol Appl* 2021;130:112466.

59.	Albrektsson T, Johansson C. Osteoinduction, osteoconduction and osseointegration. *Eur Spine J* 2001;10 Suppl 2:S96-101.

60.	Cushnie EK, Ulery BD, Nelson SJ, Deng M, Sethuraman S, Doty SB, Lo KW, Khan YM, Laurencin CT. Simple signaling molecules for inductive bone regenerative engineering. *PLoS One* 2014;9:e101627.

61.	Arnold AM, Holt BD, Daneshmandi L, Laurencin CT, Sydlik SA. Phosphate graphene as an intrinsically osteoinductive scaffold for stem cell-driven bone regeneration. *Proc Natl Acad Sci U S A* 2019;116:4855-4860.

62.	Wilson BJ, Owston HE, Iqbal N, Giannoudis PV, McGonagle D, Pandit H, Philipose Pampadykandathil L, Jones E, Ganguly P. In Vitro Osteogenesis Study of Shell Nacre Cement with Older and Young Donor Bone Marrow Mesenchymal Stem/Stromal Cells. *Bioengineering* 2024;11.

63.	O'Neill E, Awale G, Daneshmandi L, Umerah O, Lo KW. The roles of ions on bone regeneration. *Drug Discov Today* 2018;23:879-890.

64.	Kaiser F, Schroter L, Stein S, Kruger B, Weichhold J, Stahlhut P, Ignatius A, Gbureck U. Accelerated bone regeneration through rational design of magnesium phosphate cements. *Acta Biomater* 2022;145:358-371.

65.	Fuchs A, Kreczy D, Bruckner T, Gbureck U, Stahlhut P, Bengel M, Hoess A, Nies B, Bator J, Klammert U, Linz C, Ewald A. Bone regeneration capacity of newly developed spherical magnesium phosphate cement granules. *Clin Oral Investig* 2022;26:2619-2633.

66.	Bavya Devi K, Lalzawmliana V, Saidivya M, Kumar V, Roy M, Kumar Nandi S. Magnesium Phosphate Bioceramics for Bone Tissue Engineering. *Chem Rec* 2022;22:e202200136.

67.	Gelli R, Ridi F. An Overview of Magnesium-Phosphate-Based Cements as Bone Repair Materials. *J Funct Biomater* 2023;14.

68.	Babaie E, Lin B, Bhaduri SB. A new method to produce macroporous Mg-phosphate bone growth substitutes. *Mater Sci Eng C Mater Biol Appl* 2017;75:602-609.

69.	Zhao Z, Li G, Ruan H, Chen K, Cai Z, Lu G, Li R, Deng L, Cai M, Cui W. Capturing Magnesium Ions via Microfluidic Hydrogel Microspheres for Promoting Cancellous Bone Regeneration. *ACS Nano* 2021;15:13041-13054.

70.	Kazakova G, Safronova T, Golubchikov D, Shevtsova O, Rau JV. Resorbable Mg(2+)-Containing Phosphates for Bone Tissue Repair. *Materials (Basel)* 2021;14.

71.	Mammoli F, Castiglioni S, Parenti S, Cappadone C, Farruggia G, Iotti S, Davalli P, Maier JAM, Grande A, Frassineti C. Magnesium Is a Key Regulator of the Balance between Osteoclast and Osteoblast Differentiation in the Presence of Vitamin D(3). *Int J Mol Sci* 2019;20.

72.	Wu J, Liu F, Wang Z, Liu Y, Zhao X, Fang C, Leung F, Yeung KWK, Wong TM. The Development of a Magnesium-Releasing and Long-Term Mechanically Stable Calcium Phosphate Bone Cement Possessing Osteogenic and Immunomodulation Effects for Promoting Bone Fracture Regeneration. *Front Bioeng Biotechnol* 2021;9:803723.

73.	Majidinia M, Sadeghpour A, Yousefi B. The roles of signaling pathways in bone repair and regeneration. *Journal of cellular physiology* 2018;233:2937-2948.

74.	Hung C-C, Chaya A, Liu K, Verdelis K, Sfeir C. The role of magnesium ions in bone regeneration involves the canonical Wnt signaling pathway. *Acta biomaterialia* 2019;98:246-255.

75.	Lin Y-H, Lee AK-X, Ho C-C, Fang M-J, Kuo T-Y, Shie M-Y. The effects of a 3D-printed magnesium-/strontium-doped calcium silicate scaffold on regulation of bone regeneration via dual-stimulation of the AKT and WNT signaling pathways. *Biomaterials Advances* 2022;133:112660.

76.	Yu L, Gao T, Li W, Yang J, Liu Y, Zhao Y, He P, Li X, Guo W, Fan Z. Carboxymethyl chitosan-alginate enhances bone repair effects of magnesium phosphate bone cement by activating the FAK-Wnt pathway. *Bioactive Materials* 2023;20:598-609.

77.	Xiong A, He Y, Gao L, Li G, Liu S, Weng J, Wang D, Zeng H. The fabrication of a highly efficient hydrogel based on a functionalized double network loaded with magnesium ion and BMP2 for bone defect synergistic treatment. *Materials Science and Engineering: C* 2021;128:112347.

78.	Liu T, Li Y, Zhang Y, Zhao M, Wen Z, Zhang L. A biodegradable, mechanically tunable micro‐arc oxidation AZ91D‐based composite implant with calcium phosphate/chitosan coating promotes long‐term bone tissue regeneration. *Biotechnology Journal* 2021;16:2000653.

79.	Wu J, Shen Y, Wang P, Guo Z, Bai J, Wang X, Chen D, Lin X, Tang C. Self-Healing Micro Arc Oxidation and Dicalcium Phosphate Dihydrate Double-Passivated Coating on Magnesium Membrane for Enhanced Bone Integration Repair. *ACS Biomaterials Science & Engineering* 2024.

80.	Liu W, Li T, Yang C, Wang D, He G, Cheng M, Wang Q, Zhang X. Lithium-incorporated nanoporous coating formed by micro arc oxidation (MAO) on magnesium alloy with improved corrosion resistance, angiogenesis and osseointegration. *Journal of biomedical nanotechnology* 2019;15:1172-1184.

81.	Hu H, Zhao P, Liu J, Ke Q, Zhang C, Guo Y, Ding H. Lanthanum phosphate/chitosan scaffolds enhance cytocompatibility and osteogenic efficiency via the Wnt/β-catenin pathway. *Journal of Nanobiotechnology* 2018;16:1-13.

82.	Wu C, Han P, Xu M, Zhang X, Zhou Y, Xue G, Chang J, Xiao Y. Nagelschmidtite bioceramics with osteostimulation properties: material chemistry activating osteogenic genes and WNT signalling pathway of human bone marrow stromal cells. *Journal of Materials Chemistry B* 2013;1:876-885.

83.	Sun X, Jiao X, Wang Z, Ma J, Wang T, Zhu D, Li H, Tang L, Li H, Wang C. Polydopamine-coated 3D-printed β-tricalcium phosphate scaffolds to promote the adhesion and osteogenesis of BMSCs for bone-defect repair: mRNA transcriptomic sequencing analysis. *Journal of Materials Chemistry B* 2023;11:1725-1738.

84.	Xu D, Wang C, Wu J, Fu Y, Li S, Hou W, Lin L, Li P, Yu D, Zhao W. Effects of low-concentration graphene oxide quantum dots on improving the proliferation and differentiation ability of bone marrow mesenchymal stem cells through the Wnt/β-catenin signaling pathway. *ACS omega* 2022;7:13546-13556.

85.	He Y, Chen G, Li Y, Li Y, Yi C, Zhang X, Li H, Zeng B, Wang C, Xie W. Effect of magnetic graphene oxide on cellular behaviors and osteogenesis under a moderate static magnetic field. *Nanomedicine: Nanotechnology, Biology and Medicine* 2021;37:102435.

86.	Kang E-S, Kim H, Han Y, Cho Y-W, Son H, Luo Z, Kim T-H. Enhancing osteogenesis of adipose-derived mesenchymal stem cells using gold nanostructure/peptide-nanopatterned graphene oxide. *Colloids and Surfaces B: Biointerfaces* 2021;204:111807.

87.	He Y, Li Y, Chen G, Wei C, Zhang X, Zeng B, Yi C, Wang C, Yu D. Concentration‐dependent cellular behavior and osteogenic differentiation effect induced in bone marrow mesenchymal stem cells treated with magnetic graphene oxide. *Journal of Biomedical Materials Research Part A* 2020;108:50-60.

88.	Li Z, Xiang S, Lin Z, Li EN, Yagi H, Cao G, Yocum L, Li L, Hao T, Bruce KK, Fritch MR, Hu H, Wang B, Alexander PG, Khor KA, Tuan RS, Lin H. Graphene oxide-functionalized nanocomposites promote osteogenesis of human mesenchymal stem cells via enhancement of BMP-SMAD1/5 signaling pathway. *Biomaterials* 2021;277:121082.

89.	Arnold AM, Holt BD, Tang C, Sydlik SA. Phosphate modified graphene oxide: Long–term biodegradation and cytocompatibility. *Carbon* 2019;154:342-349.

90.	Daneshmandi L. Graphene-based Materials for Bone Regenerative Engineering. In. *Biomedical Engineering*. UConn Library PhD Dissertations: University of Connecticut; 2020.

91.	Nelson C, Khan Y, Laurencin CT. Nanofiber–microsphere (nano-micro) matrices for bone regenerative engineering: a convergence approach toward matrix design. *Regenerative biomaterials* 2014;1:3-9.

92.	Chen X, Fan H, Deng X, Wu L, Yi T, Gu L, Zhou C, Fan Y, Zhang X. Scaffold structural microenvironmental cues to guide tissue regeneration in bone tissue applications. *Nanomaterials* 2018;8:960.

93.	Guo W, Quan P, Fang L, Cun D, Yang M. Sustained release donepezil loaded PLGA microspheres for injection: Preparation, in vitro and in vivo study. *Asian Journal of Pharmaceutical Sciences* 2015;10:405-414.

94.	Fei F, Yao H, Wang Y, Wei J. Graphene Oxide/RhPTH(1-34)/Polylactide Composite Nanofibrous Scaffold for Bone Tissue Engineering. *Int J Mol Sci* 2023;24.

95.	Liu H, Slamovich EB, Webster TJ. Less harmful acidic degradation of poly(lacticco-glycolic acid) bone tissue engineering scaffolds through titania nanoparticle addition. *Int J Nanomedicine* 2006;1:541-5.

96.	Hussein AS, Abdullah N, Ahmadun FR. In vitro degradation of poly (D, L-lactide-co-glycolide) nanoparticles loaded with linamarin. *IET Nanobiotechnol* 2013;7:33-41.

97.	Goods JB, Sydlik SA, Walish JJ, Swager TM. Phosphate functionalized graphene with tunable mechanical properties. *Adv Mater* 2014;26:718-23.

98.	Cerna Nahuis LE, Alvim Valente C, de Freitas Oliveira D, de Souza Basso NR, Antonio Malmonge J. Preparation and Characterization of Polymeric Microfibers of PLGA and PLGA/PPy Composite Fabricated by Solution Blow Spinning. *Macromolecular Symposia* 2019;383.

99.	Makadia HK, Siegel SJ. Poly Lactic-co-Glycolic Acid (PLGA) as Biodegradable Controlled Drug Delivery Carrier. *Polymers (Basel)* 2011;3:1377-1397.

100.	Perron JK, Naguib HE, Daka J, Chawla A, Wilkins R. A study on the effect of degradation media on the physical and mechanical properties of porous PLGA 85/15 scaffolds. *J Biomed Mater Res B Appl Biomater* 2009;91:876-886.

101.	Ramchandani M, Pankaskie M, Robinson D. The influence of manufacturing procedure on the degradation of poly(lactide-co-glycolide) 85:15 and 50:50 implants. *Journal of Controlled Release* 1997;43:161-173.

102.	Tong L, Shi G, Liu Q, Qian Z, Li J, Zhang K, Zhu Y, Fang Y, Sha L, Bai L, Li Y, Wang X, Ma Y, Jirigala E, Wang H, Li X. Fabrication and evaluation of 3D printed PLGA/nHA/GO scaffold for bone tissue engineering. *Sci Rep* 2025;15:12446.

103.	Xu TO, Kim HS, Stahl T, Nukavarapu SP. Self-neutralizing PLGA/magnesium composites as novel biomaterials for tissue engineering. *Biomed Mater* 2018;13:035013.

104.	Burmester A, Willumeit-Romer R, Feyerabend F. Behavior of bone cells in contact with magnesium implant material. *J Biomed Mater Res B Appl Biomater* 2017;105:165-179.

105.	Rui S, Kubota T, Ohata Y, Yamamoto K, Fujiwara M, Takeyari S, Ozono K. Phosphate promotes osteogenic differentiation through non-canonical Wnt signaling pathway in human mesenchymal stem cells. *Bone* 2022;164:116525.

106.	Shih YR, Hwang Y, Phadke A, Kang H, Hwang NS, Caro EJ, Nguyen S, Siu M, Theodorakis EA, Gianneschi NC, Vecchio KS, Chien S, Lee OK, Varghese S. Calcium phosphate-bearing matrices induce osteogenic differentiation of stem cells through adenosine signaling. *Proc Natl Acad Sci U S A* 2014;111:990-5.

107.	Fu C, Yang X, Tan S, Song L. Enhancing Cell Proliferation and Osteogenic Differentiation of MC3T3-E1 Pre-osteoblasts by BMP-2 Delivery in Graphene Oxide-Incorporated PLGA/HA Biodegradable Microcarriers. *Sci Rep* 2017;7:12549.

108.	Soleymani H, Moghaddam MM, Naderi-Manesh H, Taheri RA. Single-layer graphene oxide nanosheets induce proliferation and Osteogenesis of single-cell hBMSCs encapsulated in Alginate Microgels. *Sci Rep* 2024;14:25272.

109.	Yue Y, Liu L, Liu P, Li Y, Lu H, Li Y, Zhang G, Duan X. Cardamonin as a potential treatment for melanoma induces human melanoma cell apoptosis. *Oncology Letters* 2020;19:1393-1399.

110.	Díaz-Tocados JM, Herencia C, Martínez-Moreno JM, Montes de Oca A, Rodríguez-Ortiz ME, Vergara N, Blanco A, Steppan S, Almadén Y, Rodríguez M. Magnesium Chloride promotes Osteogenesis through Notch signaling activation and expansion of Mesenchymal Stem Cells. *Scientific reports* 2017;7:7839.

111.	Hung CC, Chaya A, Liu K, Verdelis K, Sfeir C. The role of magnesium ions in bone regeneration involves the canonical Wnt signaling pathway. *Acta Biomater* 2019;98:246-255.

112.	Yoshizawa S, Brown A, Barchowsky A, Sfeir C. Magnesium ion stimulation of bone marrow stromal cells enhances osteogenic activity, simulating the effect of magnesium alloy degradation. *Acta Biomater* 2014;10:2834-42.

113.	Li J, Ding J, Zhou T, Li B, Wang J, Wang H, Fu L. A novel functionally graded bilayer membrane with excellent barrier function and in vivo osteogenesis promotion for guided bone regeneration. *Front Pharmacol* 2024;15:1453036.

114.	Andrew TW, Koepke LS, Wang Y, Lopez M, Steininger H, Struck D, Boyko T, Ambrosi TH, Tong X, Sun Y, Gulati GS, Murphy MP, Marecic O, Tevlin R, Schallmoser K, Strunk D, Seita J, Goodman SB, Yang F, Longaker MT, Yang GP, Chan CKF. Sexually dimorphic estrogen sensing in skeletal stem cells controls skeletal regeneration. *Nat Commun* 2022;13:6491.

115.	Rude RK, Gruber HE, Wei LY, Frausto A, Mills BG. Magnesium deficiency: effect on bone and mineral metabolism in the mouse. *Calcif Tissue Int* 2003;72:32-41.

116.	Orchard TS, Larson JC, Alghothani N, Bout-Tabaku S, Cauley JA, Chen Z, LaCroix AZ, Wactawski-Wende J, Jackson RD. Magnesium intake, bone mineral density, and fractures: results from the Women's Health Initiative Observational Study. *Am J Clin Nutr* 2014;99:926-33.

117.	Chang J, Yu D, Ji J, Wang N, Yu S, Yu B. The Association Between the Concentration of Serum Magnesium and Postmenopausal Osteoporosis. *Front Med (Lausanne)* 2020;7:381.

118.	Na D, Tao G, Shu-Ying L, Qin-Yi W, Xiao-Li Q, Yong-Fang L, Yang-Na O, Zhi-Feng S, Yan-Yi Y. Association between hypomagnesemia and severity of primary hyperparathyroidism: a retrospective study. *BMC Endocr Disord* 2021;21:170.

119.	Kocyigit SE, Katipoglu B. Hypomagnesemia may be related to frailty, gait and balance problems, and basic activities of daily living in older adults. *Acta Clin Belg* 2024;79:160-167.

120.	Wang Y, Qin B, Xia G, Choi SH. FDA's Poly (Lactic-Co-Glycolic Acid) Research Program and Regulatory Outcomes. *AAPS J* 2021;23:92.

121.	Andrews JPM, Joshi SS, Tzolos E, Syed MB, Cuthbert H, Crica LE, Lozano N, Okwelogu E, Raftis JB, Bruce L, Poland CA, Duffin R, Fokkens PHB, Boere AJF, Leseman D, Megson IL, Whitfield PD, Ziegler K, Tammireddy S, Hadjidemetriou M, Bussy C, Cassee FR, Newby DE, Kostarelos K, Miller MR. First-in-human controlled inhalation of thin graphene oxide nanosheets to study acute cardiorespiratory responses. *Nat Nanotechnol* 2024.

122.	Murphy CM, O'Brien FJ. Understanding the effect of mean pore size on cell activity in collagen-glycosaminoglycan scaffolds. *Cell Adh Migr* 2010;4:377-81.

123.	Loh QL, Choong C. Three-dimensional scaffolds for tissue engineering applications: role of porosity and pore size. *Tissue Eng Part B Rev* 2013;19:485-502.

124.	Dorcemus DL, Kim HS, Nukavarapu SP. Gradient scaffold with spatial growth factor profile for osteochondral interface engineering. *Biomed Mater* 2021;16.
