# Supplementary material for: Magnesium phosphate functionalized graphene oxide and PLGA composite matrices with enhanced mechanical and osteogenic properties for bone regeneration
Source: Regen Biomater. 2025 Jul 26;12:rbaf074. doi: 10.1093/rb/rbaf074 (PMC12364435; doi:10.1093/rb/rbaf074)
Supplement: rbaf074_Supplementary_Data [file rbaf074_supplementary_data.zip › rbaf074_Supplement_Data.zip!/TarajeW_Regen Biomat Supporting Information.docx]

**Magnesium Phosphate Functionalized Graphene Oxide and PLGA Composite Matrices with Enhanced Mechanical and Osteogenic Properties for Bone Regeneration**

Taraje Whitfield^1,3,6^, Fatemeh S. Hosseini^1,3,6^, Jason D. Orlando^7^, Chenyun Deng^7,8^, Kevin W.-H. Lo^1,2,4,10^, Ho-Man Kan^1^, Debolina Ghosh^1^, Stefanie A. Sydlik^7-9*^, Cato T. Laurencin^1-5*^

*^1^The Cato T. Laurencin Institute for Regenerative Engineering, University of Connecticut, Farmington, CT, 06030, USA.*

*^2^Department of Biomedical Engineering, University of Connecticut, Storrs, CT, 06269, USA.*

***^3^****Department of Orthopaedic Surgery, University of Connecticut Health, Farmington, CT, 06030, USA.*

***^4^****Department of Materials Science & Engineering, University of Connecticut, Storrs, CT, 06269, USA. USA.*

***^5^****Department of Chemical & Bimolecular Engineering, University of Connecticut, Storrs, CT, 06269, USA.*

*^6^Department of Skeletal Biology and Regeneration, UConn Health, Farmington, CT, 06030 USA*

*^7^Department of Chemistry, Carnegie Mellon University, Pittsburgh, PA, 15213, USA*

*^8^Department of Biomedical Engineering, Carnegie Mellon University, Pittsburgh, PA, 15213, USA*

*^9^Department of Materials Science and Engineering, Carnegie Mellon University, Pittsburgh, PA, 15213, USA*

*^10^Department of Medicine, Division of Endocrinology, University of Connecticut Health Center, School of Medicine, Farmington, CT, USA*

****Corresponding Authors:***

*Cato T. Laurencin, M.D., Ph.D.*

*E‐mail:* [*laurencin@uchc.edu*](mailto:laurencin@uchc.edu)

*Stefanie A. Sydlik, Ph.D.*

*Email:* [*ssydlik@andrew.cmu.edu*](mailto:ssydlik@andrew.cmu.edu)

**Competing Interest Statement:**

The authors have no relevant financial or non-financial interests to disclose.

**Keywords:**

Magnesium Phosphate, Graphene Oxide, Bone Regeneration, Wnt/β-Catenin, Induceron

**This File Includes:**

Supplementary Figures 1-4

Supplementary Table 1

**Appendix**

**Supplementary Figure 1: Magnesium Phosphate Graphene Synthesis and Structure**

**Supplementary Figure 2: X-Ray Photoelectron Spectroscopy (XPS) of MgPG**

**Supplementary Table 1: MgPG Bond Presence and Polyphosphate Length**

The values in Supplementary Table 1 are derived from the XPS experiment in Supplementary Figure 2. The formula below was used to calculate the average polyphosphate length within MgPG in the table above.

Furthermore, MgPG encapsulation efficacies of PLGA/MgPG1 and PLGA/MgPG5 composite matrices by PLGA were calculated based on the measurements taken from the thermogravimetric analysis (TGA) (Figure 3A).

Additionally, the matrix weight data (shown on the left from Figure 1E) allowed us to calculate the concentration of Mg^2+^ that is theoretically released from the PLGA/MgPG composite matrices (middle column).

**Supplementary Figure 3: Alizarin Red S Assay Under Growth Media Conditions**

Under growth media conditions for 14, 21, and 28 days, MgPG at 5 wt% displayed significantly higher levels of calcium deposition than PLGA. Furthermore, MgPG at 1 and 5 wt% demonstrated significantly higher levels of calcium deposition at D28 than PLGA. Quantitative experimental data were presented as mean ± standard deviation (SD). Significant levels are determined at *p < 0.05, **p < 0.01, ***p < 0.001, and ****p < 0.0001.

**Supplementary Figure 4: Future Application for MgPG Matrices**

Schematic showing a future application of the PLGA/MgPG composite microsphere-based matrices for bone regenerative engineering: implanting into a critical-sized defects in humans for seamless bone regeneration in areas that would otherwise not have any healing.
